# Encyclopedic tumor analysis for guiding treatment of advanced, broadly refractory cancers: results from the RESILIENT trial

**DOI:** 10.18632/oncotarget.27188

**Published:** 2019-09-24

**Authors:** Rajnish Nagarkar, Darshana Patil, Timothy Crook, Vineet Datta, Sagar Bhalerao, Sonal Dhande, Vijay Palwe, Shirsendu Roy, Prakash Pandit, Ashwini Ghaisas, Raymond Page, Harjeetsingh Kathuria, Ajay Srinivasan, Dadasaheb Akolkar

**Affiliations:** ^1^ HCG Manavata Cancer Centre, Nasik, India; ^2^ Datar Cancer Genetics Limited, Nasik, India; ^3^ St. Luke’s Cancer Center, Royal Surrey County Hospital, Guildford, UK; ^4^ Worcester Polytechnic Institute, Worcester, USA

**Keywords:** precision oncology, encyclopedic tumor analysis, personalized cancer treatment, objective response rate, progression free survival

## Abstract

RESILIENT (CTRI/2018/02/011808) was a single arm, open label, phase II/III study to test if label agnostic therapy regimens guided by Encyclopedic Tumor Analysis (ETA) can offer meaningful clinical benefit for patients with relapsed refractory metastatic (r/r-m) malignancies. Patients with advanced refractory solid organ malignancies where disease had progressed following ≥2 lines of systemic treatments were enrolled in the trial. Patients received personalized treatment recommendations based on integrational comprehensive analysis of freshly biopsied tumor tissue and blood. The primary end points were Objective Response Rate (ORR), Progression Free Survival (PFS) and Quality of Life (QoL). Objective Response (Complete Response + Partial Response) was observed in 54 of 126 patients evaluable per protocol (ORR = 42.9%; 95% CI: 34.3%–51.4%, *p* < 0.0001). At study completion, Disease Control (Complete Response + Partial Response + Stable Disease) was observed in 114 out of 126 patients evaluable per protocol (CBR = 90.5%; 95% CI: 83.9% - 95.0%, *p* < 0.00001) and Disease Progression in 12 patients. Median duration of follow-up was 138 days (range 31 to 379). Median PFS at study termination was 134 days (range 31 to 379). PFS rate at 90 days and 180 days were 93.9% and 82.5% respectively. The study demonstrated that tumors have latent vulnerabilities that can be identified via integrational multi-analyte investigations such as ETA. This approach identified viable treatment options that could yield meaningful clinical benefit in this cohort of patients with advanced refractory cancers.

## INTRODUCTION

It has been popularly believed [1] that analyzing the molecular structure of cancer would yield definitive strategies and therapeutic direction for improved outcomes. However, translation of this seemingly axiomatic deduction into meaningful improvements in systemic therapy has proved to be persistently elusive. While platforms and solutions for molecular analysis of tumors have become ubiquitous, widespread adoption of treatment strategies based on evidence of molecular hallmarks appears to be stymied for want of definitive data and lack of demonstrable, quantifiable clinical benefits.

Targeted treatments have been confined to their labelled indications and efforts at replication of therapeutic benefit in an organ-agnostic setting [2, 3] appear to be limited, the most notable example yet being of the checkpoint inhibitor Pembrolizumab, which was recently approved for solid organ malignancies with mismatch repair deficiency (dMMR). It is also pertinent to mention the pan-cancer drug Larotrectinib [4] which has been approved for use across solid organ malignancies with neurotrophic receptor tyrosine kinase (NTRK) gene fusion, and Tisotumab vedotin [5], which has shown promise in clinical trials to treat all tumors that express tissue factor.

There have been other [6–13] efforts to improve outcomes in hard to treat cancers using a putative correlation between molecular analysis and treatment selection in off-label or organ agnostic settings. However, these studies were either based on univariate analysis of biomarkers and/or constrained in design by restricting inclusion to patients who were positive for a predefined molecular feature of the tumor. Most of these studies [6, 7, 9] also treated patients with single agents selected on the basis on available molecular indications, even in instances where multiple drug indications may have been available. Though there is evidence [14–18] from prior trials that multi-drug combinations of cytotoxic and targeted agents may yield improved therapeutic benefit, fewer prior studies [8, 11, 12] appear to have evaluated multi-drug combinations based on tumor molecular profiling. As a consequence of these restrictions, the outcomes reported in prior precision medicine trials (such as the ones enlisted above) have either fallen short of expectations [6] or have merely suggested equivocal to incremental improvements in efficacy [7–12], and have indicated the need for further evaluation of molecular guided therapy selection approach. Only the IPREDICT Study has reported a significantly higher ORR [13].

Presently, no evidence exists on treatment strategies for r/r m-cancers based on comprehensive, multi-analyte molecular analysis with synchronous *in vitro* chemo-sensitivity profiling, in a label-agnostic manner. Accordingly, we designed the RESILIENT Study, where label-and organ-agnostic treatment strategies for patients with r/r m-cancers were based on an integrative, multi-analyte Encyclopedic Tumor Analysis (ETA) which captures in depth information about the multi-layered tumor interactome. In the RESILIENT Study, participants received personalized multi-drug therapy recommendations based on inputs from multiple molecular biomarkers as well as *in vitro* chemosensitivity testing on viable tumor cells. We present the study outcomes which demonstrate the efficacy of ETA-guided treatment options which target latent vulnerabilities of the tumor to afford meaningful clinical benefit to patients.

## RESULTS

### Patients

**Figure 1:**
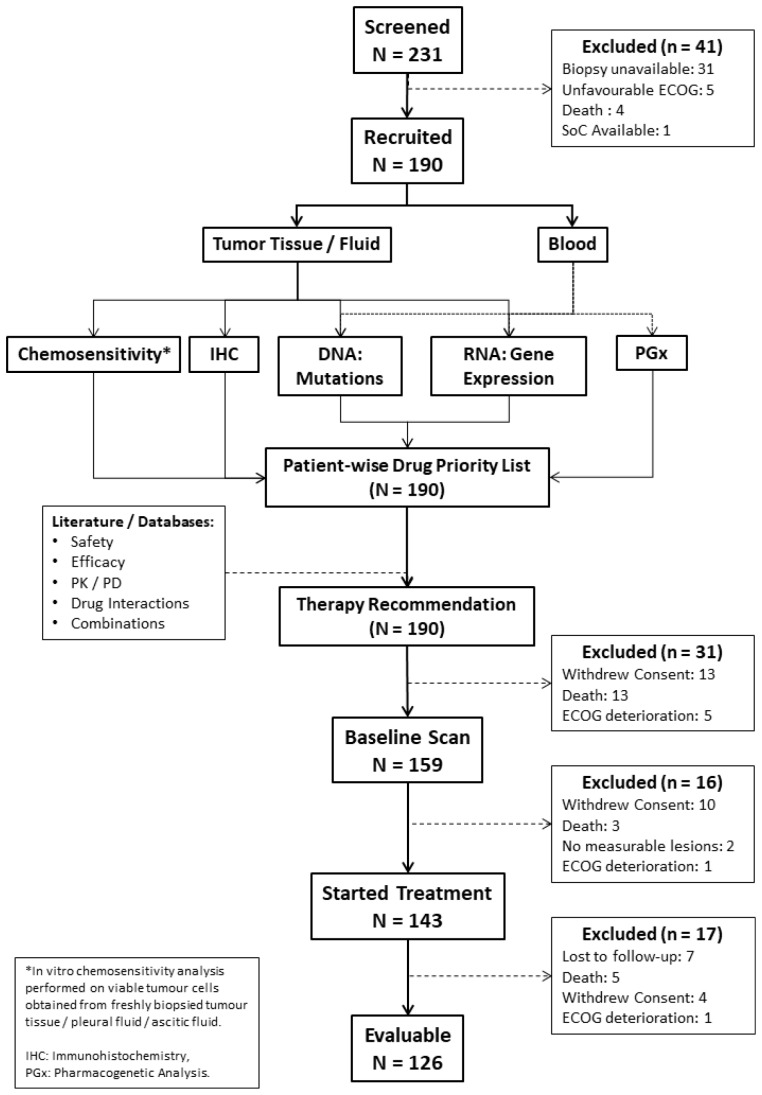
CONSORT diagram.

Between December 2017 and October 2018, 231 patients were screened for recruitment, of whom, 190 patients were recruited and 143 patients eventually started treatment as per ETA; 47 patients were excluded prior to start of treatment for various reasons including withdrawal of consent (*n =* 23), death (*n =* 16), deterioration of Eastern Cooperative Oncology Group (ECOG) performance status (*n =* 6) or unavailability of lesions measurable on a CT/PET-CT scan (*n =* 2). Treatment for the first patient commenced on January 05, 2018 and for the most recent patient on November 16, 2018. Out of the 143 patients who started treatment, 17 patients were excluded prior to any follow-up evaluation for various reasons including patient being lost to follow-up (*n =* 7), death (*n =* 5), withdrawal of consent (*n =* 4) and deterioration of health (*n =* 1). A total of 126 patients were evaluable as per study criteria and 65 patients were continuing treatment in accordance to the TR as on the lock-in date of January 25, 2019. The CONSORT diagram (Figure 1) depicts the study structure and flow. Patient demographics, cancer types and prior treatments are indicated in Table 1 with expanded and additional details in Supplementary Table 1 and Supplementary Table 2 Patient-wise extent of disease and sites of metastases are indicated in Supplementary Table 3. The distribution of cancer types among the study population was an accurate representation of the locoregional prevalence rates [19].

**Table 1:** Baseline characteristics of Intent to Treat (ITT) and evaluable patients

Parameter	ITT	Evaluable
	Number (%)	Number (%)
**Ethnicity**		
South Asian (Indian)	143 (100%)	126 (100%)
**Gender**		
Male	73 (51.0%)	65 (51.6%)
Female	70 (49.0%)	61 (48.4%)
**Age**		
Min	25	24
Max	75	72
Median	50	50
**Cancer Types and Organs**		
Bone	4 (2.1%)	3 (2.4%)
Breast	26 (18.2%)	21 (16.7%)
Cervical	5 (3.5%)	5 (3.9%)
Colorectal	14 (9.8%)	14 (11.1%)
Oesophagus	2 (1.4%)	2 (1.6%)
Gastric	7 (4.9%)	6 (4.8%)
Head and Neck	36 (25.2%)	31 (24.6%)
Hepatobiliary	7 (4.9%)	6 (4.8%)
Kidney	4 (2.8%)	4 (3.2%)
Lung	7 (4.9%)	5 (4.0%)
Neuroendocrine tumors	3 (2.1%)	3 (2.4%)
Ovarian	9 (6.3%)	8 (6.3%)
Pancreatic	8 (5.6%)	8 (6.3%)
Prostate	1 (0.7%)	1 (0.8%)
Sarcoma	5 (3.5%)	4 (3.2%)
Skin	3 (1.8%)	3 (2.4%)
Testes	2 (1.2%)	2 (1.6%)
**Grade of Tumor**		
1 (Well-differentiated)	13 (9.1%)	11 (8.7%)
2 (Moderately differentiated)	54 (37.8%)	50 (39.7%)
3 (Poorly differentiated/undifferentiated)	52 (36.4%)	43 (34.1%)
(Grade unevaluable)	24 (16.8%)	22 (17.5%)
**Total Prior Lines of Therapy**		
1–2	38 (26.6%)	36 (28.6%)
3–4	61 (42.7%)	52 (41.3%)
≥ 5	44 (30.8%)	38 (30.2%)

### Landscape of genomic alterations

**Figure 2:**
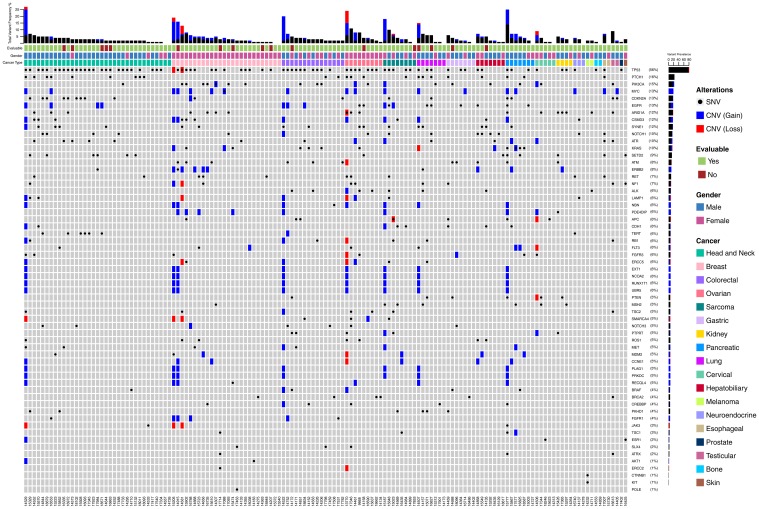
Landscape of genomic alterations in the Intent to Treat (ITT) population. Each vertical column indicates a single patient (5-digit numeric identifier in the bottom X-axis). Vertically stacked grey boxes in each column indicate individual genes (gene names on right Y-axis). Black dots within each box indicates a point mutation (single nucleotide variation), whereas blue and red shaded boxes indicate gain or loss of gene copy respectively. Patients are grouped according to cancer types – colour coded boxes immediately above the grey stacked boxes. Gender is indicated above the cancer type. Patients who were evaluable per protocol are indicated in the topmost row of colour-coded boxes. Bar graph on the top indicates combined variant frequency (%) per patient. Bar graph to the right indicates total frequency of occurrence of alterations in that particular gene in the ITT population.

Figure 2 depicts the landscape of genomic alterations in the Intent to Treat (ITT) population. Point mutations in TP53 were most frequently encountered (56%) gene variations in the ITT population, which also included two instances of copy loss. Similarly, point mutations in PTCH1 (16%) and PIK3CA (15%) were the second and third most frequently encountered variants. Gain of gene copy was observed most often in MYC, ERBB2, NBN, PDE4DIP, EXT1, NCOA2, RUNX1T1, UBR5, CCNE1, PLAG1, PRKDC and RECQL4. Loss of gene copy was most frequently encountered in genes such as ARID1A, KRAS, ATM, NF1, LAMP1, APC, RB1, FLT3, FGFR3, ERCC5, PTEN, SMARCA4 and JAK3. Patient-wise actionable gene alterations that formed the basis for therapy selection are indicated in Supplementary Table 4. Additionally, gene expression data in terms of mRNA as well as immunohistochemistry (IHC) were also considered for therapy selection and are also indicated (where actionable) in Supplementary Table 4.

### Treatments

Among the 143 patients who received ETA-guided treatment under RESILIENT, 45 patients received combinations of cytotoxic agents, 5 patients received combinations of targeted agents and 93 patients received combinations of cytotoxic and targeted agents. Endocrine therapy agents were administered to 21 patients in addition to cytotoxic and/or targeted agents. Patients were administered treatments as per institutional protocols and treatments were continued until study completion or dose-limiting toxicity or progression or any other end-point, such as patient opting out/defaulting. Patient-wise details of prior treatments received, ETA-guided treatment combinations, and rationale for ETA guided agents are indicated in Supplementary Table 4.

### Response to treatment

**Figure 3:**
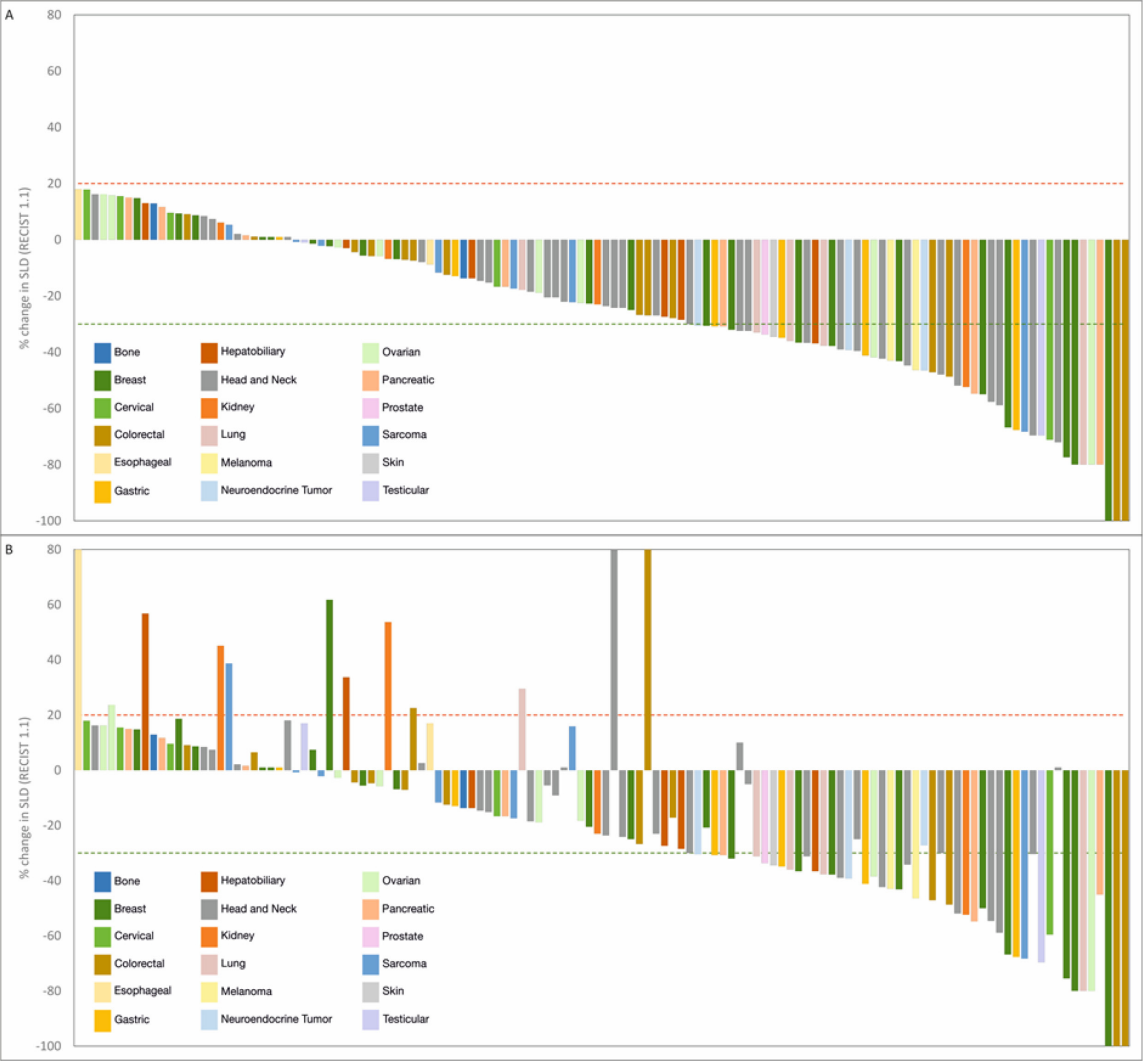
Summary of outcomes in RESILIENT. (**A**) Waterfall chart of best response. Treatment response was evaluated as per RECIST 1.1. Percent change in dimensions of target lesions (Sum of Largest Diameters, SLD) between baseline and at evaluation are graphically represented. Patients are arranged in descending order of change (%) in SLD. (**B**) Waterfall chart of response at study completion. Treatment response was evaluated as per RECIST 1.1. Percent change in dimensions of target lesions (Sum of Largest Diameters, SLD) between baseline and at evaluation are graphically represented. Sequence of patients is same as in Figure 2A to indicate change in status (if any) at study completion.

**Figure 4:**
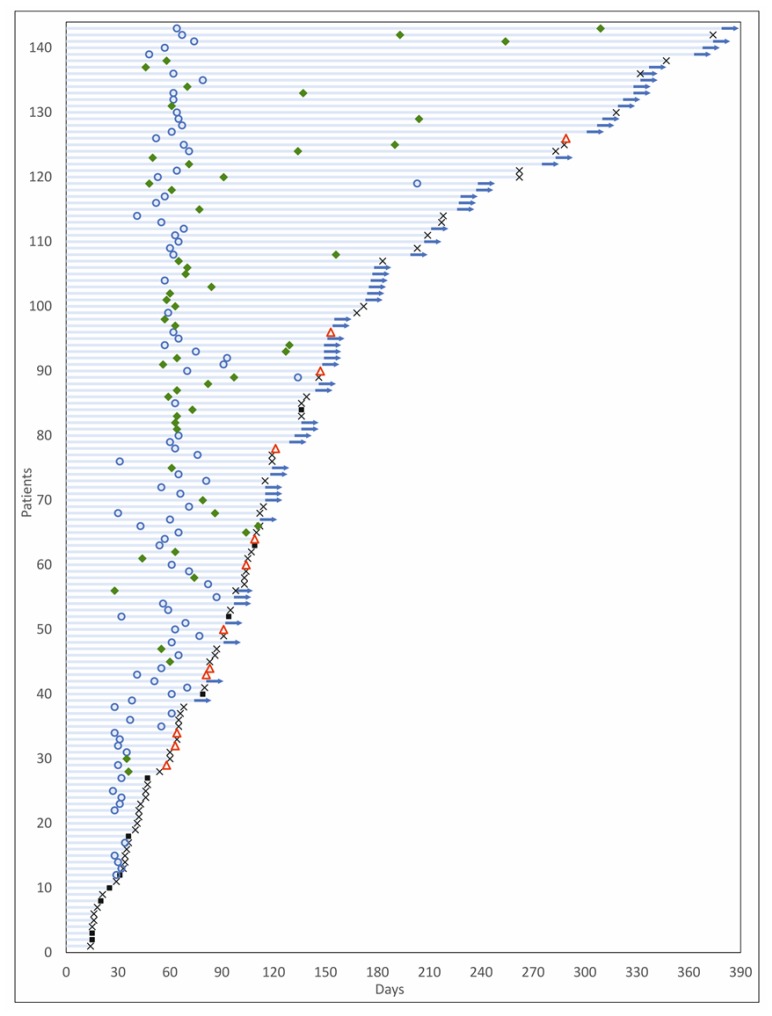
Swimmer plot of patient response. The Y-axis indicates patients while the X-axis indicates time (days).♦: Partial Response/Complete Response; Ο:Stable Disease; △: Disease Progression; ×: Lost to follow-up/Withdrew Consent; ▀: Death; ➜: Progression Free Survival. For radiological response status, only the first scan and subsequent scan where response status changed are indicated.

Among the 126 patients who underwent follow-up scans and were thus evaluable per protocol, Objective Response was observed in 54 patients (ORR = 42.9%), including 3 Complete Responses (CR) and 51 Partial Responses (PR). At study completion, 3 patients (2.4%) had continued CR, 45 patients (35.7%) had PR, 66 patients (52.4%) had Stable Disease (SD), and 12 patients (9.5%) showed Disease Progression. The Clinical Benefit Rate (CBR) was determined to be 90.5%. Response Evaluation in the Intent to Treat (ITT) population, which included 17 patients who were excluded prior to any follow-up scans, indicated an ORR of 37.8%, which was not significantly lower than the patients evaluable per protocol. Similarly, CBR was determined to be 68.5% when evaluated in the ITT population, in those patients where SD was determined to be ≥60 days. Characteristics of response are indicated in Table 2. Waterfall Charts depict the best radiological response (Figure 3A) and radiological response at study termination (Figure 3B) of all 126 patients. A Swimmer Plot (Figure 4) depicts temporal trends in response and duration of response.

**Table 2:** Clinical activity of ETA-guided therapies in patients with r/r-m solid organ malignancies

Parameter	Value
**Objective Response Rate**	
Number of patients	54
% of cohort (95% CI)	42.9 (34.3–51.5)
*P* value	<0.00001
**Status at Study Completion**	
Complete Response (%)	3 (2.4%)
Partial Response (%)	45 (35.7%)
Stable Disease (%)	66 (52.4%)
Disease Progression (%)	12 (9.5%)
**Time to Objective Response (days)**	
Median	64
Range	28–309
**Duration of Follow-Up (days)**	
Median	138
Range	31–379
**Progression Free Survival (days)**	
Median	134
Range	31–379

### Progression free survival (PFS)

**Figure 5:**
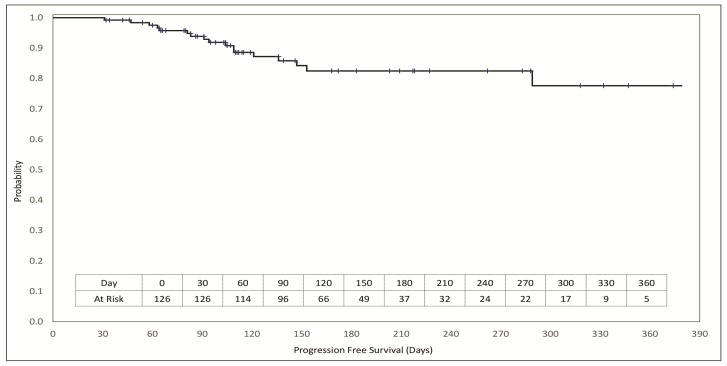
Kaplan Meier plot of progression free survival. Patients at risk at each milestone are indicated in the inset table. Vertical cross-bars indicate censoring events.

Patients were followed up for a median duration of 138 days (range 31 to 379 days). The Kaplan Meier plot of PFS is depicted in Figure 5. PFS rates at 90 days and at 180 days were 93.9% and 82.5% respectively. A comparison of PFS on RESILIENT (PFS2) with that on last prior systemic line of treatment (PFS1) for the relevant patient was ascertainable as per trial criteria (PFS1 ≤90 days) in 62 patients where median PFS1 was 72 days (range 22 to 111) and PFS2 was 120 days (range 34 to 374). Of these 62 patients, 22 patients (35.5%) achieved a PFS2: PFS1 ratio of ≥2.5 and 47 patients (75.8%) achieved and PFS2: PFS1 ratio of >1.3.

### Metastases

Among the evaluable patients, (*n =* 126), the most commonly observed sites of metastases at baseline were lymph nodes (*n =* 84, 66.7%), lungs (*n =* 35, 27.8%), bones (*n =* 30, 23.8%) and liver (*n =* 31, 24.6%). Central nervous system involvement as brain metastases was observed in 9 patients (7.9%) and bone marrow involvement was observed in 3 patients (2.4%). Presence or absence of metastases in vital organs such as brain, lung or liver did not appear to impact outcomes in response to ETA-guided therapy; ORR or CBR in patients with brain, lung or liver metastases were not found to be significantly different from patients who did not have metastases to these organs (Supplementary Table 5). Significantly, all brain metastases were observed to be stable (*n =* 7) or had regressed (*n =* 2) at the most recent evaluation for these patients; none of the patients reported new or increase in size of brain metastases. At RESILIENT Study completion, 12 patients (9.5%) had progressed, among whom 9 patients (7.1%) progressed with no new distant metastases which were observed only in the other 3 patients (2.4%).

### Therapy related adverse events

Adverse Events (AEs) were recorded as per the National Cancer Institute - Common Terminology Criteria for Adverse Events (NCI-CTCAE) v5.0 [20] (Table 3). All the 143 patients in the Intent to Treat (ITT) population were evaluated for therapy-related AEs. Onset of therapy related AEs was observed approximately up to 1 week, post therapy and time to resolution ranged between 1 to 2 weeks. The most common AEs (any grade) reported in ≥10% patients were Fatigue, Anorexia, Mucositis Oral, Edema, Diarrhoea, Pyrexia, Neutropenia, Myalgia, Vomiting, Anemia, Constipation, Thrombocytopenia and Pruritis/Rash. The only grade 3 AE in ≥10% patients was neutropenia (11.3%). Haematological toxicities of any grade were observed in 47 patients (32.9%) while grade 3 haematological toxicities were observed in 28 patients (19.6%). Patients with metastases to bone marrow (2.4%) did not appear to be at greater risk of haematological therapy-related toxicities as compared to the entire cohort. Overall, grade 3 and above therapy-related AEs were reported in 57 patients (39.9%) among whom dose readjustment or interruptions were necessitated in 47 (32.9%) patients due to Anemia, Edema, Hypotension, increased blood bilirubin, Mucositis Oral, Neutropenia, Thrombocytopenia and Vomiting. No grade 4 treatment-related AEs were reported in any of the patients. There were no mortalities that could be ascribed to treatments received. Owing to the patients receiving unique combinations of treatment agents, as well as individualized management of dosage and schedule, there were no discernible patterns in adverse events (or categories of adverse events) that could be ascribed to specific mechanistic classes (e. g., TKI/platins) or categories (e. g., cytotoxic/targeted/endocrine) of drugs. All AEs were managed by administration of standard of care agents or procedures as required.

**Table 3:** Therapy-related adverse events in intent to treat population

Adverse events	Any grade		Grade ≥3	
	No of patients	%	No of patients	%
Fatigue	121	84.6%	9	6.3%
Anorexia	92	64.3%	6	4.2%
Mucositis Oral	57	39.9%	13	9.1%
Edema	39	27.3%	4	2.8%
Pyrexia	35	24.5%	8	5.6%
Diarrhoea	35	24.5%	1	0.7%
Neutropenia	32	22.4%	16	11.2%
Myalgia	30	21.0%	3	2.1%
Vomiting	26	18.2%	6	4.2%
Anemia	22	15.4%	12	8.4%
Constipation	20	14.0%	1	0.7%
Thrombocytopenia	18	12.6%	12	8.4%
Pruritis/Rash	16	11.2%	1	0.7%
Nausea	14	9.8%	2	1.4%
Peripheral neuropathy	11	7.7%	2	1.4%
Pain at site of biopsy	9	6.3%	3	2.1%
Hyper-/Hypotension	8	5.6%	6	4.2%
Alopecia	6	4.2%	0	0.0%
Increased blood bilirubin	4	2.8%	3	2.1%
Eletrolyte Imbalance	3	2.1%	2	1.4%
Hoarseness	2	1.4%	1	0.7%
Pneumonitis	2	1.4%	1	0.7%
Dysuria	1	0.7%	0	0.0%
**Any Event**	**143**	**100%**	**57**	**39.9%**

### Quality of Life (QoL)

Quality of Life was measured based on a brief questionnaire that evaluated the patients’ functional and symptomatic status which are innately linked to the ECOG status. Patients’ feedback was obtained on functional, symptomatic and overall health status at baseline and at most recent follow-up or at study termination. 83.9% patients indicated stable to improved functional status, 74.2% patients indicated stable to decreased symptomatic status and 90.3% patients indicated stable to improved overall health status.

## DISCUSSION

Data from RESILIENT shows that r/r m-cancers have unexplored vulnerabilities amenable to treatment. Consequently, it is possible to obtain durable objective response and disease control in a significant proportion of the total patient population, by guiding treatments based on ETA. Contrary to the discouraging or equivocal data from previous studies, the ORR and CBR observed in RESILIENT demonstrates the clinical impact of ETA and potential benefits or label- and organ-agnostic therapy selection in clinical practice.

The discouraging outcomes of prior trials have been often used as a benchmark for vocal skepticism [21, 22] and to dissuade label-agnostic individualized treatment selection. A comparison of RESILIENT with 6 such widely reviewed studies/trials is hence relevant (Supplementary Table 6). The SHIVA trial [6] targeting 3 molecular pathways with Molecular Targeting Agents (MTAs) reported weak outcomes and went on to discourage the use of MTAs outside their current indications. The MyPathway trial [7] reported ORR of 23% across 14 different tumor types. A pilot study by Von Hoff et al [8] showed PFS ratio of ≥1.3 in 27% of patients treated with cytotoxic and targeted/endocrine agents based on limited molecular profiling. The MOSCATO trial [9] reported ORR of 11% in patients following molecular profiling and treatment with cytotoxic, targeted and endocrine therapies. The M. D. Anderson Cancer Center reported [10] an ORR of 27% in a retrospective analysis of several Phase-I clinical trials in personalized medicine where a limited set of molecular aberrations were targeted using approved cytotoxic, targeted and investigational agents. Interim data from 4 arms of the NCI – MATCH [11] study showed an aggregate ORR of 7.5% among patients who received targeted therapy based on molecular changes. More recently, the IPREDICT study [12] was significant in that it indicated an ORR of 45% and 75% of patients indicated a potential gain in PFS by ~30% based on molecularly matched treatments. The WINTHER study [13] reported an ORR of 13% and 9% respectively in the two arms where patients were treated on the basis of molecular features in DNA and RNA respectively.

While these studies relied on univariate molecular marker analysis for therapy selection, they also generally had restrictive inclusion criteria which recruited only those patients who had a predefined molecular target for treatment with preselected choice of agents. This inclusion qualification rate was factored in for an indexed comparison between results of some of these trials [6–11] and RESILIENT (Supplementary Table 7) to evaluate the parameters of the various studies which necessitated bias correction in their reported ORRs. The advantage of an ETA-guided approach to therapy selection, as in RESILIENT, is evident in absence of prequalifying molecular features, due to which potentially all patients with solid organ malignancies stand to benefit rather than just the limited proportion of the real-world patient population where tumors harbor the predefined feature. Analysis of differentially (over) expressed genes based on mRNA or IHC provided additional therapy options for several patients, including those where actionable gene alterations were unavailable. As opposed to the WINTHER trial [13] where actionable information from DNA and RNA competed with each other for efficacy analysis, they were complementary to one another in the RESILIENT protocol. Similarly, *in vitro* chemosensitivity analysis using viable tumor cells provided direct functional evidence of drug efficacy which aided therapy selection. There have been concerns about the suitability of *in vitro* chemosensitivity analysis for therapy selection [23] in cancers based on outcomes of prior studies. However, prior efforts appear to be based on single agents and regimens included in Standard of Care (SoC) for the cancer types. On the other hand, we evaluated a comprehensive panel of FDA-approved agents in an organ agnostic setting based on which optimum agent (s) were selected. Thus, the outcomes in RESILIENT were superior to the next line SoC treatment options and indicated the possibility of viable alternatives to checkpoint inhibitors [24–28] (Supplementary Table 8) for representative cancer types.

The durability of response in RESILIENT, in terms of the 90-day PFS rate, appears to be significant as deduced from Kaplan Meier plots (Supplementary Table 9). It is generally believed that PFS decreases with every subsequent line of therapy. Thus, contemporary precision oncology trials have benchmarked PFS (while on trial) to the last failed systemic line of treatment to determine therapeutic advantage. While a 30% increase has been indicated as significant, outcomes of RESILIENT show that it is possible to achieve significant (2.5×) increase in PFS as compared to the last line. A significant number of patients were progression free at study completion, and hence the median reported PFS in RESILIENT reflects the status at study completion and not the final outcome. Duration of Response (DoR) and Overall Survival (OS) are presently not mature for reporting.

Most of the study population had experienced progression of disease with new distant metastases of the last failed treatment, whereas on the RESILIENT protocol, appearance of distant metastases on progression was well controlled; out of the 12 patients where progression was seen, local progression was observed in 9 patients while only 3 patients presented new distant metastases. The suppression of metastatic tendency of the disease is significant and its impact cannot be overemphasized in view of the shifting appreciation of late stage disease management as recognized in Prostate cancer where Metastasis Free Survival (MFS) has been described as a relevant clinical trial endpoint [29].

Though objective response is a desirable aim at every treatment threshold, an equally relevant consideration for treatment of advanced refractory cancers is achievement of stable disease (SD) with associated improvements not only in time-dependent end points but in quality of life measures. In this respect, the disease control achieved in RESILIENT is clearly encouraging.

Though there have been several reports [14–18] of de novo combinations of targeted and cytotoxic agents yielding improved therapeutic benefits, patients in most prior studies received single agents. Only a few precision medicine studies included combination treatments which were administered to all (or limited set of) patients based on molecular profiling. The MD Anderson Study [10] retrospectively evaluated patients from several drug trials where patients received single agents and combinations of approved as well as experimental agents. In case of the WINTHER trial [13], though genomic and transcriptomic molecular marker data were evaluated, majority of patients were treated with single agents based on either DNA or RNA in the respective study arms. In the RESILIENT Study, all patients received combinations of cytotoxic, targeted or endocrine agents based on cellular and molecular evidence evaluated by the ETA. The authors seek to draw attention of the reader to the fact that none of the study patients received experimental or unapproved drugs. All patient-specific therapy combinations recommended through the ETA included only those drugs that have already been approved for treatment of (same/different) cancers with well characterized toxicity profiles. Thus, AEs were well controlled even in this heavily pre-treated population with tumor evolution and systemic deterioration following multiple prior lines of treatments. Notably, there were no grade 4 treatment related AEs in RESILIENT; for comparison a prior meta-analysis of Phase I trials between 2001 to 2012 of cytotoxic drugs reported Grade 4 AEs in 19.9% in patients [30].

Having discussed the benefits of ETA, an insight into the limitations is also pertinent. ETA requires fresh tissue from a *de novo* biopsy tissue where the quality and quantity of biopsied tissue could be of concern. Patients who have progressed on multiple lines of treatment are often psychologically fatigued for further invasive procedures and possible hospitalization. The impact of previous treatments on the overall health of the patients, especially on the bone marrow reserve can impede compliance with ETA guided treatments. However, adoption of ETA-guided approach at an earlier treatment stage could obviate limitations associated with less beneficial SoC treatments. In the ITT population (*n =* 143), among the 17 patients who were excluded prior to any follow-up, 6 patients were lost to follow-up due to inability to travel from other cities for treatment. Similarly, among the 17 patients who were excluded after the first evaluation, 12 patients were lost to follow-up for the same reason. Due to these early exclusions, the median follow-up duration appears underrepresented in the study population. As a corrective action, for all subsequent enrolments into RESILIENT, priority and preference were given to patients living within the same city, or within a reasonable distance with access to direct transportation. Another significant impediment towards achieving improved outcomes was the non-availability of USFDA or EMA approved treatment agents for incorporation in the TR, as several such drugs are not approved in India and possibly in several other countries. RESILIENT is also confined to the south Asian – Indian population, although it seems unlikely that the outcomes would vary across ethnicities.

## METHODS

All laboratory processes were conducted at a CAP and ILAC accredited institution.

### Study design

RESILIENT was a single arm, single centre, non-randomized phase II/III prospective trial for evaluation of treatment response to therapy based on ETA recommendation in r/r m-cancers patients. The Ethics Committees of the participating institutes had approved the trial. The design of the trial acknowledged the rationale [8] that owing to the diversity in cancer types and unique treatment history of each patient, there can be no accurate external control for each patient. Therefore, rather than a randomised two arm trial design, a single arm design would more accurately evaluate and represent treatment benefits from the ETA guided approach, when benchmarked against the patients last (failed) line of treatment. Thus, prior treatment response of patients served as the virtual control arm [8]. The Progression Free Survival (PFS) on ETA-guided treatment (PFS2) was benchmarked against that (PFS1) on the last (failed) line of systemic treatment in those patients where PFS1 was ≤ 90 days.

### Patients

RESILIENT recruited patients with solid organ malignancies who had either failed at least two prior lines of Standard of Care (SoC) treatments or where SoC treatment options were unavailable or further unviable. Eligible patients had radiologically evident and measurable disease, an Eastern Co-operative Oncology Group (ECOG) performance status of ≤2 and who consented to provide tissue and blood/fluid samples. Patients who fulfilled the above criteria were counselled regarding the potential benefits and risks of the trial. Thereafter, willing patients provided duly signed, informed consents. The complete eligibility criteria are available at http://apps.who.int/trialsearch/Trial2.aspx?TrialID=CTRI/2018/02/011808.

### Encyclopedic tumor analysis (ETA)

Tumors employ myriad mechanisms, feedback loops and redundancies at each of the functional layers of coding, transcription, regulation and protein synthesis, such as reactivation of signalling pathways, cross-talk between various pathways, post-translational modification, heterogeneity of tumors, clonal evolution of resistant variants and more [31, 32]. Consequently, each functional layer influences the processes towards sustaining survival and proliferation singularly and cumulatively. Thus, any drug - feature conjugation that looks towards a single layer of the process will inevitably miss the interactions and context in the other layers of this interactome. The ETA captures and contextualizes data from multi-layered tumor interactome, including *in vitro* response/resistance of viable cells. Individual procedures as part of the ETA are described in the sub-sections below.

### Tissue and blood collection

Approximately 5 × 5 × 5 mm freshly biopsied tumor tissue was transferred into 5 mL transport medium and stored at 4°C during transit. Fresh tissue was either processed immediately or cryopreserved at −80° C.

10 mL peripheral blood was collected by venous puncture in Cell-Free DNA BCT^®^ and EDTA vacutainer tubes. Blood was stored and transported at 4° C. Plasma was separated by centrifugation at 3000× *g* for 20 min at 4° C, followed by 16000× *g* for 10 min at 20–25° C. Plasma samples without hemolysis were processed immediately.

### Histopathology and immunohistochemistry

Formalin-Fixed Paraffin-Embedded (FFPE) blocks were prepared as per standard procedures. Histopathological (HPE) and immunohistochemical (IHC) analyses were carried out as per standard procedures. Tumor content of freshly biopsied tissue was determined by HPE evaluations. Tissue samples with ≥80% tumor content were considered as acceptable for molecular evaluations.

### DNA isolation

Genomic DNA was isolated from fresh tissue samples using the PureLink^®^ Genomic DNA Mini Kit and MagMAX FFPE DNA isolation kit (Thermo Fisher Scientific, USA) as per the manufacturer’s instructions. DNA was quantified at 260 nm and quality was determined by measuring the ratio of absorbance at 260/280 nm using a NanoDrop 2000 (Thermo Fisher Scientific, Waltham, USA).

Total ctDNA was purified from 2 mL plasma using a Circulating Nucleic Acid kit (QIAGEN, Germantown, USA) as per the manufacturer’s protocol. ctDNA was quantified using an HS DNA Qubit assay (Life Technologies, Carlsad, USA).

### RNA isolation

Total tumor RNA was isolated from fresh tumor tissue by using mirVana miRNA isolation kit (Ambion, Austin, USA) as per the manufacturer’s instruction. Total RNA was quantified using a Qubit 2 Fluorometer (Thermo Fisher Scientific, Waltham, USA) with the manufacturer’s RNA assay kit.

RNA from exosomes were isolated from peripheral blood plasma. Plasma samples (2 ml) from EDTA tubes were centrifuged at 16000× *g* for 10 min at 4° C and filtered via a 0.45 μm membrane to remove larger vesicles. The filtrate was used for the extraction of total exosomal RNA using an ExoRNeasy serum/plasma kit (QIAGEN, Germantown, USA) according to the manufacturer’s protocol [17]. Purified exosomal RNA was quantified using an miRNA Qubit assay (Life Technologies, Carlsad, USA).

### Tumor DNA profiling

Tumor DNA was sequenced for 453 genes using Oncomine Comprehensive Assay v3 and Ion AmpliSeq Comprehensive Cancer Panel (Thermo Fisher, USA) as per user recommended protocols. Briefly, 40 ng DNA was used for NGS library preparation via PCR-based Ampliseq target enrichment protocol. Libraries of 100 pmol were sequenced using Ion Proton (Thermo Fisher Scientific, Waltham, USA). Torrent Suite™ v5.2 (Thermo Fisher Scientific, Waltham, USA) software was used to perform primary analysis, including signal processing and base calling. Primary QC parameters were: minimum read length of 25 bases, read quality trimming of 17 QV, window size for quality trimming 30 bp. The processed sequenced data were aligned to the reference genome GRCh37/hg19 to generate Binary Alignment/Map (BAM) files. Sequencing data were considered for downstream analysis with coverage at ≥10,000× depth and >80% amplicons with at least 600 reads. The aligned data were analyzed using Torrent Variant Caller software with optimized parameters such as minimum allele frequency (0.003), minimum mapping quality (4), minimum coverage (600), down sample to coverage (10,000) and position bias (1). Reported somatic variants of >0.5% allele frequency (AF) were compared to the reference genome hg19. The Integrative Genomics Viewer (IGV) was used to visualize the read alignment and the presence of variants against the reference genome and to confirm the veracity of the variant calls by checking for possible strand biases and sequencing errors. All the germline variants found in the 1000 Genomes Project or The Exome Aggregation Consortium (ExAC) with a frequency of >0.1% were excluded. All somatic mutations were annotated, sorted and interpreted using COSMIC and/or TCGA data. Variants with <0.5% AF were confirmed orthogonally with digital droplet polymerase chain reaction (ddPCR, BioRad) using the rare mutation assay as per the manufacturer’s protocol.

### Targeted whole transcriptome analysis

The Ion AmpliSeq™ Transcriptome Human Gene Expression Research Panel was used to determine the expression of 20,802 genes including 18,574 coding genes and 2228 non-coding genes based on University of California Santa Cruz (UCSC) hg19 annotation. Historical RNA from normal adjacent tissue was used as a control for transcriptome analysis. A barcoded cDNA library was generated with a SuperScript^®^ VILO™ cDNA Synthesis kit from 40 ng of total RNA. The cDNA was amplified using Ion AmpliSeq™ technology as per the manufacturer’s instructions (Thermo Fisher Scientific). Amplified cDNA libraries were evaluated for quality on a Bioanalyzer 2100E using a high sensitivity DNA 1000 chip (Agilent Technologies) and quantified using an Ion Library TaqMan™ Quantitation Kit (Thermo Fischer Scientific. Pooled libraries of 100 pM were amplified using emulsion PCR on an Ion Torrent OneTouch2 and enriched as per the manufacturer’s instructions. Templated libraries were sequenced on an Ion Torrent Proton™ sequencing system, using an Ion PI sequencing kit and an Ion PI chip (Thermo Fisher Scientific). Analysis of AmpliSeq RNA sequencing data was performed using the AmpliSeq-RNA plugin available for Ion Torrent sequencing platforms. This plugin uses the Torrent Mapping Alignment Program (TMAP—https://github.com/iontorrent/TMAP), which is optimized for aligning raw sequencing reads (from Ion Torrent) against the hg19 transcriptome reference sequence against regions defined in the Browser Extensible Display (BED) file (hg19_AmpliSeq_Transcriptome_21K_v1. bed). The quality of the raw data was evaluated based on three parameters: number of reads, mean read length and target detected (% of all amplicons that had ≥10 assigned reads). Differential gene expression analysis was performed using R/Bioconductor package edgeR with raw read counts from AmpliSeq. Read count normalization was performed using the Counts Per Million (CPM). Significant differential expressed genes were called using the following threshold: absolute log fold-change ≥2 and Benjamini–Hochberg adjusted *p* < 0.05. The commercial software iPathway Guide (Advaita) was used for pathway analysis to explore significantly affected pathways.

### *In vitro* chemosensitivity profiling of viable tumor cells

Viable tumor cells were isolated from freshly biopsied tumor tissue by standard procedures and maintained *in vitro*. Viable cells were seeded into multi-well plates and allowed to adhere. Adherent viable cells *in vitro* were treated with a panel of FDA-approved anti-cancer drugs for 24 hours after which apoptotic cell death events were determined. All assays included positive and negative controls as well as untreated cell controls to determine baseline apoptotic events. Response to each drug was determined after subtracting baseline apoptosis in untreated controls. Data from all investigations were integrated to identify agents and their combinations with maximum projected efficacy and safety.

### Therapy recommendation

An interdisciplinary tumor board comprising of oncologists, pathologists, other clinicians, molecular biologists, and bioinformaticians evaluated tumor-data including somatic and germline mutations in DNA for actionable gene alterations, pharmacogenetics analysis of alterations in drug metabolism enzymes (DME), differentially expressed genes and pathways for targeting, immunohistochemistry and *in vitro* chemosensitivity profiling of viable tumor derived cells. Drug indications derived from all evaluations were integrated and harmonized to create a drug preference list based on maximum projected efficacy and identification of potential risks due to alterations in DME. All drugs in the preference list were evaluated for further safety and efficacy based on information in published literature and public databases, and included sources such as (i) safety and efficacy findings from Phase I/II/III trials and meta analyses, (ii) analyses of multi-drug combinations [14–16] including targeted-cytotoxic drug combinations, (iii) pharmacokinetic and pharmacodynamics studies, and (iii) reported drug-interactions. This approach yielded patient-specific priority list of drugs and their combinations with projected efficacy and safety profiles, i. e., the Therapy Recommendation (TR). Availability of treatment agents in India was also considered in design of patient-specific regimens. The patient-specific TRs did not exclude drugs or combinations that may have been indicated in Standard of Care (SoC) for that cancer; ETA did not aim to deny regimens merely because of indication in SoC, rather ETA sought to identify optimum regimens with putative benefit for the patient, irrespective of the empirical nature of existing guidelines.

### Treatments

Patient specific TRs were submitted to the treating clinician within 7 to 10 days of receipt of patient samples. The treating clinician evaluated the suitability of the suggested treatments and oversaw therapy administration. Being a single site study, TRs and treatments for all patients were evaluated by the Principal Investigator and other treating clinicians, due to which there were no subjective differences in interpretations for therapy management. Clinicians and patients were not blinded to the treatment. The treating clinicians exercised their discretion with regard to the optimum starting dose on a case by case basis considering patient safety, risk of therapy related adverse events (AEs), prior treatments and history of AEs. Treatments were administered/continued as per the standard practice and protocols of the treating institution until guideline-imposed limitations for duration of treatment were encountered, or until dose-limiting toxicity or disease progression or in the event of patient exclusion.

### Evaluations

The baseline status of the disease was determined by a ^18^F-Fluorodeoxyglucose Positron Emission Tomography – Computed Tomography (FDG PET-CT) scan before initiation of treatment. A baseline MRI scan was also performed to identify any brain metastases. Response was evaluated on the lines of RECIST 1.1 criteria [33] through a further scan after the patient completed at least two treatment cycles or 60 days of treatment, except in cases where the treating clinician advised evaluation in the interim. Thereafter, follow-up scans were performed every 6 to 10 weeks. Status of brain metastases was determined by follow-up MRI scans. All scans were independently reviewed by a panel of external expert radiologists.

### Patient monitoring

All study participants underwent periodic investigations such as complete blood counts, hepatic and renal function tests, urinalysis and left ventricular ejection fraction (LVEF) to determine fitness to receive or continue treatment as per study protocol. Other investigations such as ultrasonography, x-ray or endoscopy was carried out on recommendation of the treating oncologist. Adverse events were recorded during patient admissions as well as via telephonic follow-up during those weeks where patients did not visit the hospital. All adverse events were reported as per NCI-CTCAE v5 criteria. All grade 3 adverse events were flagged and followed up on a daily basis until resolution. Patients received printed instructions of subsequent treatment and imaging appointments as well as telephonic reminders. Patients had 24-hour telephonic access to study-coordinators as well as access to emergency/ambulance services.

### Endpoints

The primary efficacy end point of the study was Objective Response Rate (ORR) defined as the percentage of patients who achieved Complete Response (CR) or Partial Response (PR) during the active study phase. Other end points were Clinical Benefit Rate (CBR) and Progression Free Survival (PFS). CBR was defined as the percentage of patients who achieved CR, PR or Stable Disease (SD). PFS was defined as time from commencement of treatment under ETA to disease progression or death during the active study phase. PFS on ETA-guided treatment (PFS2) was compared against that (PFS1) on the last (failed) systemic line. The qualitative end point was Quality of Life (QoL), based on patient’s feedback on symptomatic and functional status at baseline and at study termination or most recently available follow-up.

### Statistical methods and analysis

The sample size of the study was determined on the basis of the ORR, assuming that the ORR in such refractory advanced stage cancer patients is <10%. Simon’s 2-stage design was used to validate adequacy of cohort size for assessment of ETA based therapy. The null hypothesis that the true response rate is 10% was tested against a one-sided alternative. Initially, at least 21 patients were required to accrue; if there were 2 or fewer responses, the study was required to be stopped. Else, at least 45 additional patients were required to accrue for a total minimum of 66 patients. The null hypothesis would be rejected if 11 or more responses were observed in 66 patients. With 66 evaluable patients, this design yields a type I error rate of 5% and power of 90% when the true response rate is 25%. The 95% CI of ORR was constructed using binomial distribution (Clopper-Pearson estimation method). Patient demographics were analysed with descriptive statistics. Contingency tables described the categorical data with counts and percentages. Continuous data was summarized using median and range. CONSORT diagram, waterfall plot and bar graphs were used to summarize the data. Kaplan-Meier estimator was used to estimate survival function.

## SUPPLEMENTARY MATERIALS







## Abbreviations

ETA: Encyclopedic Tumor Analysis
r/r-m: relapsed refractory metastatic
ORR: Objective Response Rate
PFS: Progression Free Survival
QoL: Quality of Life
dMMR: mismatch repair deficiency
NTRK: neurotrophic receptor tyrosine kinase
ECOG: Eastern Cooperative Oncology Group
RECIST: Response Evaluation Criteria in Solid Tumors
CONSORT: Consolidated Standards of Reporting Trials
CR: Complete Responses
PR: Partial Responses
SD: Stable Disease
CBR: Clinical Benefit Rate
AE: Adverse Events
NCI: National Cancer Institute
CTCAE: Common Terminology Criteria for Adverse Events
ITT: Intent to Treat
DoR: Duration of Response
MFS: Metastasis Free Survival
SoC: Standard of Care (SoC)
DCGL: Datar Cancer Genetics Limited
HCG-MCC: HCG Manavata Cancer Centre
CAP: College of American Pathologists
cfDNA: cell free DNA
PCR: Polymerase Chain Reaction
NGS: Next Generation Sequencing
mRNA: messenger RNA
FFPE: Formalin Fixed Paraffin Embedded
IHC: immunohistochemistry
SNP: Single Nucleotide Polymorphisms
TR: Therapy Recommendations
FDG: ^18^F-Fluorodeoxyglucose
PET-CT: Positron Emission Tomography – Computed Tomography
MRI: Magnetic Resonance Imaging
