# Supplementary material for: Encyclopedic tumor analysis for guiding treatment of advanced, broadly refractory cancers: results from the RESILIENT trial
Source: Oncotarget. 2019 Sep 24;10(54):5605–21. doi: 10.18632/oncotarget.27188 (PMC6771458; doi:10.18632/oncotarget.27188)
Supplement: Supplementary file 1 [file oncotarget-10-5605-s001.pdf]

Encyclopedic tumor analysis for guiding treatment of advanced, broadly refractory cancers: results from the RESILIENT trial

SUPPLEMENTARY MATERIALS

Supplementary Table 1: Detailed stratification of cancer types

Parameter	ITT	Evaluable
	**Number (%)**	**Number (%)**
**Bone**	4 (2.1%)	3 (2.4%)
Chordoma	1	1
Osteogenic Sarcoma	2	2
**Breast**	26 (18.2%)	21 (16.7%)
Adenocarcinoma: TNBC	8	6
Adenocarcinoma: ER+/PR-/Her2-	2	1
Adenocarcinoma: ER+/PR+/Her2-	2	2
Adenocarcinoma: ER-/PR-/Her2+	7	6
Adenocarcinoma: ER+/PR-/Her2+	1	1
Adenocarcinoma: ER+/PR+/Her2 Eq	3	2
Adenocarcinoma: ER+/PR+/Her2+	3	3
BRCA Positive (driver) among the above	*2*	*1*
**Cervical**	5 (3.5%)	5 (3.9%)
Adenocarcinoma	1	1
Squamous Cell Carcinoma	4	4
**Colorectal**	14 (9.8%)	14 (11.1%)
Colon Adenocarcinoma	6	6
Rectum Adenocarcinoma	7	7
Rectum Adenosquamous Carcinoma	1	1
KRAS, NRAS positive among the above	*3,2*	*3,2*
**Oesophagus**	2 (1.4%)	2 (1.6%)
Squamous Cell Carcinoma	2	2
Her2 positive among all of the above	*1*	*1*
**Gastric**	7 (4.9%)	6 (4.8%)
Adenocarcinoma	7	6
Her2 positive among the above	1	1
**Head and Neck**	36 (25.2%)	31 (24.6%)
Maxillary Sinus	1	1
Nasopharynx	2	2
Oral Cavity	28	23
Oropharynx	5	5
**Hepatobiliary**	7 (4.9%)	6 (4.8%)
Adenocarcinoma – Gallbladder	2	2
Cholangiocarcinoma – Bile Duct	1	1

Cholangiocarcinoma – Hepatic	1	1
Hepatocellular Adenocarcinoma	3	2
**Kidney**	4 (2.8%)	4 (3.2%)
Clear Cell Carcinoma	3	3
Renal Pelvis – Squamous Cell Carcinoma	1	1
**Lung**	7 (4.9%)	5 (4.0%)
NSCLC – Adenocarcinoma	4	4
NSCLC – Squamous Cell Carcinoma	2	1
Sarcomatoid Carcinoma	1	-
**Neuroendocrine tumors**	3 (2.1%)	3 (2.4 %)
Cervix	1	1
Occult Primary	1	1
Carcinoid Tumor - Duodenum	1	1
**Ovarian**	9 (6.3%)	8 (6.3%)
Adenocarcinoma	3	3
Serous Adenocarcinoma	2	2
Serous Cystadenocarcinoma	4	3
BRCA Positive (driver) among the above	*0*	*0*
**Pancreatic**	8 (5.6%)	8 (6.3%)
Adenocarcinoma	4	4
Periampullary Adenocarcinoma	3	3
Anaplastic Carcinoma	1	1
**Prostate**	1 (0.7%)	1 (0.8%)
Adenocarcinoma	1	1
**Sarcoma**	5 (3.5%)	4 (3.2%)
Gastrointestinal Stromal Tumor – Rectum	1	1
Leiomyosarcoma – Retroperitoneum	1	-
Leiomyosarcoma – Uterus	1	1
Liposarcoma – Retroperitoneum	1	1
Myxofibrosarcoma – Thorax	1	1
**Skin**	3 (1.8%)	3 (2.4%)
Pilomatrical carcinoma	1	1
Melanoma	2	2
**Testes**	2 (1.2%)	2 (1.6%)
Non-seminomatous germ cell tumor	1	1
Seminoma	1	1
**Metastasis**		
Lymph Node	95 (66.4%)	84 (66.7 %)
Lung	38 (26.6%)	35 (27.8 %)
Bones	35 (24.5%)	30 (23.8 %)
Liver	31 (21.7%)	31 (24.6 %)
Peritoneum	24 (16.8%)	23 (18.3 %)
Brain	13 (9.1%)	9 (7.9 %)
Soft Tissue	11 (7.7%)	8 (6.3 %)
Bone Marrow	4 (2.8%)	3 (2.4 %)

Supplementary Table 2: Prior therapy status of study cohort at baseline

Parameter	ITT	Evaluable
	Number (%)	Number (%)
**Prior Therapies Received**		
Systemic only	30 (21.0 %)	26 (20.6 %)
Systemic + Radiotherapy	22 (15.4 %)	21 (12.4 %)
Systemic + Surgery	39 (27.3 %)	34 (20.0 %)
Radiotherapy Surgery	0 (0.0 %)	0 (0.0 %)
Systemic + Radiotherapy + Surgery	52 (36.4 %)	45 (26.5 %)
**All Prior Lines of Therapy**		
1–2	38 (26.6%)	36 (28.6 %)
3–4	61 (42.7%)	52 (41.3 %)
≥ 5	44 (30.8%)	38 (30.2 %)
Minimum lines	1	1
Maximum lines	17	17
Median lines	3	3
**Prior Systemic Lines**		
1–2	81 (56.6%)	72 (57.1 %)
3–4	45 (31.5%)	39 (31.0 %)
≥ 5	17 (11.9%)	15 (11.9 %)
Minimum lines	1	1
Maximum lines	14	14
Median lines	2	2
**Prior Systemic Treatments**		
Cytotoxic	136 (95.1%)	120 (95.2 %)
Endocrine	8 (5.6%)	8 (6.3 %)
Targeted	37 (25.9%)	33 (26.2 %)
**Prior Surgeries**		
None	52 (36.4 %)	47 (37.3 %)
1	67 (46.9 %)	58 (46.0 %)
≥ 2	24 (16.8 %)	21 (16.7 %)
**Prior Irradiation**		
None	69 (48.3 %)	60 (47.6 %)
1	56 (39.2 %)	48 (38.1 %)
≥ 2	18 (12.6 %)	18 (14.3 %)

Supplementary Table 3: Patient-wise extent of disease and metastatic sites. See Supplementary Table 3

Supplementary Table 4: Patient-wise details of prior lines of treatment, ETA-guided treatments and indications for ETA-guided treatments. See Supplementary Table 4

Supplementary Table 5: Metastases, objective response rate and clinical benefit rate

Site (Organ)	Status of Metastasis	Number of patients	Objective Response Rate		Clinical Benefit Rate (Final)
			Best	Final	
Lung	Absent	91	44.0% (34.2%–54.2%)	38.5% (34.2%–54.2%)	91.2% (83.4%–95.7%)
	Present	35	37.1% (23.1%–53.7%)	34.3% (20.8%–50.9%)	88.6% (73.5%–96.1%)
Liver	Absent	95	44.2% (34.6%–54.2%)	38.9% (29.7%–49.0%)	91.6% (84.0%–95.9%)
	Present	31	35.5% (21.1%–53.1%)	32.3% (18.5%–49.9%)	87.1% (70.6%–95.5%)
Brain	Absent	117	41.0% (32.5%–50.1%)	35.9% (27.8%–44.9%)	89.7% (82.8%–94.2%)
	Present	9	55.6% (26.6%–81.2%)	55.6% (26.6%–81.2%)	100.0% (65.5%–100.0%)

ORR and CBR were evaluated in patients as described. Patients were stratified on the basis of presence or absence of metastases to lung, liver or brain at baseline (recruitment). Neither ORR nor CBR appeared to be significantly influenced by presence or absence of metastases to these organs. Figures within parentheses represent 95% Confidence Intervals (CI).

Supplementary Table 6: Study designs in perspective

Parameter	Study identifier						
	SHIVA	My Pathway	MD Anderson	MOSCATO	Von Hoff	NCI-Match	RESILIENT
	STUDY DESIGN						
Molecular Filter	Yes	Yes	Yes	Yes	Yes	Yes	No
	MOLECULAR INDICATIONS						
SNV	Y	Y	Y	Y	-	Y	Y
CNA	-	-	-	Y	-	Y	Y
Rearrangements	-	-	Y	-	-	Y	Y
Indels	-	-	-	Y	-	Y	Y
IHC	Y	Y	Y	Y	Y	-	Y
DGE	-	Y	-	Y	Y	-	Y
	LIVE TUMOR CELL ANALYSIS						
Chemo-Sensitivity	-	-	-	-	-	-	Y
	MOLECULAR COVERAGE						
Genes for DNA	21	4	11	75	-	(varying)	409
Genes for RNA	3	1	-	(RNASeq)	62	-	>20000
	THERAPY PROFILE						
Cytotoxic	-	-	Y	Y	Y	-	Y
Targeted	Y	Y	Y	Y	Y	Y	Y
Endocrine	Y	-	-	Y	Y	Y	Y
Experimental	-	-	Y	-	-	Y	-

As compared to the largely univariate indications for therapy selection in other trials, the scope of investigations in the RESILIENT trial provided a significantly more comprehensive multi-layered view of the tumor's molecular and functional landscape, which facilitated more meaningful therapy selection for patients.
SNV: Single Nucleotide Variations; CNA: Copy Number Alterations; Indels: Insertions and Deletions; IHC: Immunohistochemistry; DGE: Differential Gene Expression.

Supplementary Table 7: Impact of exclusion criteria/molecular filter on outcomes

#	Parameter	Study Identifier						
		SHIVA	My Pathway*	MD Anderson	MOSCATO	Von Hoff	NCI-Match*	RESILIENT
1	Patients Screened	741	N.R.	1283	1035	106	N.R.	231
2	Data Available (A)	496	N.R.	1144	843	86	N.R.	190
3	Molecular Filter	Yes	Yes	Yes	Yes	Yes	Yes	None
3	Treatable Patients (B)	293	251	460	411	84	N.R.	190
4	Patients Treated	195	251	211	199	66	177	143
5	Correction Factor#	0.59	0.05	0.40	0.49	0.98	0.09**	1.00
6	Evaluable Patients	195	251	211	193	66	177	126
7	ORR Reported	4.1%	23%	27%	11%	9.1%	8.1%	42.9%**
8	ORR Adjusted@	2.4%	1.15%	10.8%	5.4%	8.9%	0.73%	42.9%**

The Comparator trials in this table employed a molecular feature-based exclusion filter which enrolled only those patients where the tumor harboured pre-defined molecular features. This inclusion bias was factored in to evaluate the real-word outcome rates among the larger patient population who were initially considered for treatment. RESILIENT did not include any such exclusion filter due to which the outcome rates are unaffected.

*Interim Data. #Correction Factor = B/A (= Treatable Patients/Patients where Available). @ORR Adjusted = ORR Reported ′ Correction Factor. @CBR Adjusted = CBR reported × Correction Factor. **Observed patient accrual rate of ~9% for 10 arms. (http://ecog-acrin.org/nci-match-eay131/interim-analysis). **ORR in ITT population indicated in Results section.

Supplementary Table 8: ORR of SoC agents and checkpoint inhibitors

Cancer Type	Treatment Option/Modality	Objective Response Rate
Head and Neck	Methotexate (SoC)	3.9
	Nivolumab	13.3
	Pembrolizumab	18
	RESILIENT	45.2
Colorectal	Regorafenib (SoC)	1.0
	Nivolumab	0.0
	Pembrolizumab	0.0
	RESILIENT	35.7

After failure of initial-line therapy in advanced Head and Neck cancers (HNSCC) or Colorectal cancers (CRC), Methotrexate and Pemetrexed are respectively considered as next line chemotherapy agents. Checkpoint inhibitors approved for use in similar setting in HNSCC and CRC include Pembrolizumab and Nivolumab. All CRC in RESILIENT were proficient in mismatch repair (pMMR), i.e., Microsatellite Stable (MS-S), where single Checkpoint inhibitors are not reported to yield objective response.

Supplementary Table 9: Comparison of 90-day PFS rates across various studies

Study Identifier	Cancer Types	Intervention	PFS Rate (90-Day)
Keynote-016 (c)	Colorectal, MMR-proficient	Pembrolizumab	10
Keynote-012 (b)	Head and Neck, PD-L1 Negative	Pembrolizumab	25
SHIVA	All solid organ malignancies	Patient-specific	40
Keynote-012 (a)	Head and Neck, PD-L1 Positive	Pembrolizumab	40
Checkmate-141	Head and Neck, All PD-L1	Nivolumab	40
Checkmate-142	Colorectal, MMR-deficient	Nivolumab	65
MD Anderson	All solid organ malignancies	Patient-specific	70
Keynote-016 (b)	Non-Colorectal, MMR-deficient	Pembrolizumab	80
Keynote-016 (a)	Colorectal, MMR-deficient	Pembrolizumab	90
Resilient	All solid organ malignancies	Patient-specific	94
