# Supplementary material for: Encyclopedic tumor analysis for guiding treatment of advanced, broadly refractory cancers: results from the RESILIENT trial
Source: Oncotarget. 2019 Sep 24;10(54):5605–21. doi: 10.18632/oncotarget.27188 (PMC6771458; doi:10.18632/oncotarget.27188)
Supplement: Supplementary file 2 [file oncotarget-10-5605-s002.docx]

**Supplementary Table 3: Patient-wise extent of disease and metastatic sites in the Intent to Treat (ITT) population**

| **ID** | **Gender** | **Age** | **Cancer Type** | **Primary** | **Metastases** | | | | | | | | | |
| --- | --- | --- | --- | --- | --- | --- | --- | --- | --- | --- | --- | --- | --- | --- |
|  |  |  |  |  | **Lung** | **Liver** | **Peritoneum** | **Lymph Nodes** | **Bones** | **Bone Marrow** | **Soft Tissue** | **Brain** | **Other Organs** | **Total Sites** |
| 14553 | M | 42 | Bone | Y | Y | - | - | - | - | - | Y | - | Y | 2 |
| 14569 | M | 60 | Bone | Y | - | - | - | - | - | - | Y | - | - | 1 |
| 14859 | M | 28 | Bone | - | Y | - | - | Y | - | - | Y | - | Y | 3 |
| 14133 | F | 41 | Breast | Y | - | - | - | Y | Y | - | - | - | - | 2 |
| 14183 | F | 36 | Breast | Y | Y | Y | - | - | Y | Y | - | - | Y | 4 |
| 14207 | F | 35 | Breast | Y | - | - | - | Y | - | - | - | Y | Y | 3 |
| 14275 | F | 55 | Breast | Y | - | - | - | Y | Y | - | - | - | - | 2 |
| 14307 | F | 49 | Breast | - | Y | Y | Y | - | Y | - | - | Y | - | 4 |
| 14355 | F | 39 | Breast | - | - | Y | - | - | - | - | - | - | - | 1 |
| 14363 | F | 53 | Breast | Y | - | - | - | Y | - | - | - | Y | - | 2 |
| 14581 | F | 60 | Breast | - | - | Y | - | - | Y | - | - | - | - | 2 |
| 14656 | F | 61 | Breast | - | - | Y | - | Y | Y | Y | - | - | - | 4 |
| 14668 | F | 49 | Breast | - | Y | - | Y | Y | Y | - | - | - | Y | 5 |
| 14915 | F | 39 | Breast | - | - | - | - | Y | - | - | - | Y |  | 2 |
| 15205 | F | 46 | Breast | Y | Y | - | - | Y | Y | - | - | - | Y | 3 |
| 15372 | F | 45 | Breast | Y | - | Y | Y | Y | Y | - | - | - | Y | 5 |
| 15474 | F | 54 | Breast | Y | - | - | - | Y | Y | - | - | - | - | 2 |
| 15540 | F | 72 | Breast | - | Y | - | - | Y | - | - | - | - | Y | 2 |
| 15610 | F | 39 | Breast | Y | - | - | - | Y | Y | - | - | Y | - | 3 |
| 15723 | F | 53 | Breast | y | - | Y | - | Y | - | - | - | - | - | 2 |
| 15902 | F | 47 | Breast | - | Y | Y | - | Y | - | - | - | Y | - | 3 |
| 16735 | F | 54 | Breast | - | Y | - | - | Y | Y | - | - | - | Y | 3 |
| 16788 | F | 60 | Breast | Y | Y | - | - | Y | - | - | - | Y | - | 2 |
| 17088 | F | 41 | Breast | Y | - | Y | - | Y | - | - | - | - | - | 2 |
| 17093 | F | 68 | Breast | - | Y | Y | - | Y | Y | - | - | - | Y | 4 |
| 17463 | F | 45 | Breast | Y | - | - | - | Y | Y | - | - | Y | - | 3 |
| 17714 | F | 75 | Breast | Y | Y | - | - | Y | Y | - | - | - | Y | 3 |
| 18038 | F | 28 | Breast | Y | - | - | - | Y | Y | - | - | Y | - | 3 |
| 18848 | F | 31 | Breast | - | - | Y | - | - | Y | - | - | - | - | 2 |
| 18246 | F | 60 | Cervix | Y | - | - | - | - | - | - | - | - | Y | 1 |
| 14137 | F | 35 | Cervix | Y | Y | Y | Y | - | - | - | - | - | Y | 3 |
| 14313 | F | 65 | Cervix | Y | Y | Y | Y | - | - | - | - | - | Y | 3 |
| 14506 | F | 59 | Cervix | Y | - | - | - | - | Y | - | - | - | - | 1 |
| 15623 | F | 44 | Cervix | Y | - | - | Y | - | - | - | - | - | - | 1 |
| 17344 | F | 54 | Cervix | Y | Y | - | - | Y | - | - | - | - | Y | 2 |
| 14172 | F | 55 | Colorectal | - | - | - | Y | Y | - | - | Y | - | Y | 4 |
| 14851 | F | 39 | Colorectal | - | Y | Y | - | - | - | - | - | Y | - | 2 |
| 17459 | F | 28 | Colorectal | Y | - | Y | - | Y | - | - | - | - | - | 2 |
| 17508 | M | 47 | Colorectal | - | - | - | Y | Y | - | - | - | - | - | 2 |
| 17531 | F | 34 | Colorectal | - | - | - | Y | Y | - | - | - | - | - | 2 |
| 17782 | M | 33 | Colorectal | Y | - | - | Y | - | - | - | - | - | - | 1 |
| 17798 | M | 45 | Colorectal | Y | - | - | Y | Y | Y | - | - | - | Y | 5 |
| 14152 | F | 24 | Colorectal | Y | - | - | - | Y | - | Y | - | - | - | 2 |
| 14171 | M | 64 | Colorectal | - | Y | - | - | Y | - | - | Y | - | Y | 3 |
| 14206 | F | 58 | Colorectal | - | Y | Y | - | Y | - | - | - | - | Y | 3 |
| 14226 | F | 32 | Colorectal | Y | - | - | - | - | - | - | - | - | Y | 1 |
| 14522 | M | 43 | Colorectal | Y | Y | - | - | Y | - | - | - | - | Y | 2 |
| 14563 | M | 36 | Colorectal | Y | Y | - | - | - | - | - | - | - | Y | 1 |
| 14752 | M | 61 | Colorectal | Y | Y | - | - | Y | - | - | - | - | Y | 2 |
| 14899 | M | 70 | Colorectal | - | Y | Y | - | - | Y | - | - | - | Y | 3 |
| 18834 | M | 60 | Colorectal | Y | Y | - | - | Y | - | - | - | - | Y | 2 |
| 14278 | M | 52 | Duodenum | Y | - | - | - | Y | - | - | - | - | - | 1 |
| 15730 | F | 66 | Esophageal | Y | - | - | - | Y | - | - | - | - | - | 1 |
| 17007 | M | 62 | Esophageal | Y | - | - | - | Y | - | - | - | - | - | 1 |
| 14688 | M | 56 | Gastric | Y | - | - | Y | - | - | - | - | - | Y | 2 |
| 14364 | M | 52 | Gastric | Y | - | - | - | Y | Y | - | - | - | - | 2 |
| 14402 | M | 69 | Gastric | Y | - | - | - | Y | - | - | - | - | - | 1 |
| 14459 | F | 27 | Gastric | - | - | - | - | Y | - | - | - | - | - | 1 |
| 16486 | M | 56 | Gastric | Y | - | Y | - | Y | - | - | - | - | - | 2 |
| 16714 | F | 54 | Gastric | - | - | - | Y | Y | - | - | - | - | - | 2 |
| 18096 | M | 55 | Gastric | - | - | - | Y | - | - | - | - | - | Y | 2 |
| 14533 | F | 57 | Head and Neck | Y | - | - | - | - | - | - | - | - | Y | 1 |
| 14557 | F | 49 | Head and Neck | Y | - | - | - | - | - | - | - | - | Y | 1 |
| 18093 | M | 45 | Head and Neck | Y | Y | Y | - | Y | Y | - | - | - | Y | 4 |
| 14252 | M | 51 | Head and Neck | Y | - | - | - | - | - | - | - | - | Y | 1 |
| 15132 | M | 45 | Head and Neck | Y | - | - | - | - | - | - | - | - | Y | 1 |
| 15570 | M | 49 | Head and Neck | Y | - | - | - | - | - | - | - | - | Y | 1 |
| 15648 | M | 51 | Head and Neck | Y | - | - | - | - | - | - | - | - | Y | 1 |
| 15928 | M | 41 | Head and Neck | Y | - | - | - | - | - | - | - | - | Y | 1 |
| 16553 | M | 36 | Head and Neck | Y | - | - | - | - | - | - | - | - | Y | 1 |
| 16972 | M | 50 | Head and Neck | Y | - | - | - | - | - | - | - | - | Y | 1 |
| 18571 | M | 54 | Head and Neck | Y | - | - | - | - | - | - | - | - | Y | 1 |
| 14295 | F | 55 | Head and Neck | Y | - | - | - | Y | - | - | - | - | - | 1 |
| 14529 | M | 35 | Head and Neck | Y | - | - | - | Y | - | - | - | - | - | 1 |
| 14552 | M | 50 | Head and Neck | Y | - | - | - | Y | - | - | - | - | - | 1 |
| 14573 | F | 46 | Head and Neck | Y | - | - | - | Y | - | - | - | - | - | 1 |
| 14644 | M | 55 | Head and Neck | Y | - | - | - | Y | Y | - | - | - | - | 2 |
| 14710 | M | 47 | Head and Neck | Y | - | - | - | Y | - | - | - | - | - | 1 |
| 14726 | M | 54 | Head and Neck | - | - | - | - | Y | - | - | - | - | Y | 2 |
| 14844 | M | 47 | Head and Neck | Y | - | - | - | Y | - | - | - | - | - | 1 |
| 15852 | M | 46 | Head and Neck | Y | - | - | - | Y | - | - | - | - | - | 1 |
| 16093 | M | 42 | Head and Neck | Y | Y | - | - | Y | - | - | - | - | Y | 2 |
| 16335 | M | 42 | Head and Neck | Y | - | - | - | Y | - | - | - | - | - | 1 |
| 16863 | M | 49 | Head and Neck | Y | Y | - | - | - | - | - | - | - | Y | 1 |
| 17345 | M | 43 | Head and Neck | - | - | - | - | Y | - | - | - | - | - | 1 |
| 17417 | M | 38 | Head and Neck | Y | - | - | - | Y | - | - | - | - | - | 1 |
| 17488 | M | 35 | Head and Neck | Y | Y | - | - | - | - | - | - | - | Y | 1 |
| 17540 | M | 57 | Head and Neck | Y | Y | - | - | - | - | - | - | - | Y | 1 |
| 17984 | M | 47 | Head and Neck | Y | - | - | - | Y | - | - | - | - | - | 1 |
| 18011 | M | 38 | Head and Neck | Y | - | - | - | Y | - | - | - | - | - | 1 |
| 18102 | M | 46 | Head and Neck | - | - | - | - | Y | - | - | - | - | - | 1 |
| 18679 | M | 37 | Head and Neck | Y | - | - | - | Y | - | - | - | - | - | 1 |
| 15223 | M | 42 | Head and Neck | Y | - | - | - | - | - | - | - | - | Y | 1 |
| 17334 | F | 61 | Head and Neck | Y | - | - | - | - | - | - | - | - | Y | 1 |
| 15329 | M | 51 | Head and Neck | Y | - | - | - | Y | - | - | - | - | - | 1 |
| 17733 | M | 45 | Head and Neck | Y | - | - | - | Y | - | - | - | - | - | 1 |
| 17923 | M | 66 | Head and Neck | Y | Y | - | - | Y | - | - | - | - | Y | 2 |
| 14858 | F | 67 | Hepatobiliary | - | Y | Y | Y | Y | - | - | - | - | Y | 4 |
| 15549 | F | 32 | Hepatobiliary | Y | - | Y | - | Y | Y | - | - | - | - | 3 |
| 18435 | F | 53 | Hepatobiliary | - | Y | Y | - | Y | Y | - | - | - | Y | 4 |
| 14135 | M | 57 | Hepatobiliary | Y | - | - | - | - | - | - | - | - | Y | 1 |
| 15292 | M | 58 | Hepatobiliary | Y | - | - | - | - | - | - | - | - | Y | 1 |
| 16129 | M | 70 | Hepatobiliary | - | - | Y | Y | Y | - | - | - | - | - | 3 |
| 18617 | F | 71 | Hepatobiliary | Y | - | - | - | Y | - | - | - | - | Y | 2 |
| 14264 | F | 59 | Kidney | - | - | Y | - | - | Y | - | - | - | - | 2 |
| 15297 | M | 43 | Kidney | Y | Y | - | - | Y | Y | - | - | - | Y | 5 |
| 15426 | M | 62 | Kidney | Y | - | - | Y | Y | - | - | - | - | Y | 3 |
| 17365 | M | 33 | Kidney | - | - | - | - | Y | - | - | - | - | - | 1 |
| 16827 | M | 57 | Lung | Y | - | - | - | - | - | - | - | Y | - | 1 |
| 15312 | F | 46 | Lung | Y | - | - | - | - | - | - | - | Y | - | 1 |
| 14157 | M | 55 | Lung | Y | Y | - | - | Y | Y | - | - | - | Y | 3 |
| 14173 | M | 62 | Lung | Y | - | Y | - | Y | - | - | - | - | - | 2 |
| 14507 | M | 43 | Lung | - | - | - | - | Y | Y | Y | Y | - | - | 4 |
| 15071 | M | 46 | Lung | Y | - | - | - | - | - | - | - | Y | - | 1 |
| 17901 | M | 59 | Lung | Y | - | - | - | Y | - | - | - | - | - | 1 |
| 14232 | M | 56 | Melanoma | Y | - | - | Y | Y | - | - | - | - | Y | 4 |
| 14711 | F | 46 | Melanoma | - | Y | - | Y | Y | Y | - | - | - | Y | 7 |
| 16740 | M | 62 | Occult Primary | - | - | Y | - | Y | - | - | - | - | - | 2 |
| 9668 | F | 48 | Ovarian | Y | ? | - | Y | Y | - | - | Y | - | - | 3 |
| 13770 | F | 50 | Ovarian | Y | - | - | - | - | - | - | Y | - | - | 1 |
| 14134 | F | 52 | Ovarian | - | - | Y | - | Y | - | - | - | - | - | 2 |
| 14158 | F | 35 | Ovarian | Y | Y | - | - | Y | Y | - | - | - | Y | 3 |
| 14461 | F | 61 | Ovarian | - | - | - | - | Y | - | - | Y | - | - | 2 |
| 15637 | F | 62 | Ovarian | Y | - | - | - | Y | - | - | - | - | Y | 2 |
| 16590 | F | 65 | Ovarian | - | - | - | Y | - | Y | - | - | - | - | 2 |
| 17346 | F | 50 | Ovarian | - | - | - | Y | Y | - | - | - | - | - | 2 |
| 18662 | F | 64 | Ovarian | - | - | - | Y | Y | - | - | - | - | - | 2 |
| 17577 | M | 70 | Pancreatic | Y | - | - | - | - | - | - | - | - | Y | 1 |
| 14727 | F | 69 | Pancreatic | Y | - | Y | - | Y | Y | - | - | - | Y | 4 |
| 15003 | F | 69 | Pancreatic | Y | - | - | - | - | Y | - | - | - | - | 1 |
| 15187 | F | 34 | Pancreatic | Y | - | - | - | Y | - | - | - | - | - | 1 |
| 15777 | M | 59 | Pancreatic | Y | - | - | - | Y | - | - | - | - | - | 1 |
| 15867 | M | 55 | Pancreatic | - | - | - | - | Y | - | - | - | - | - | 1 |
| 16825 | M | 63 | Pancreatic | Y | - | Y | - | - | - | - | - | - | - | 1 |
| 18587 | M | 49 | Pancreatic | Y | Y | Y | - | Y | - | - | - | - | Y | 3 |
| 17402 | M | 57 | Prostate | y | - | - | - | Y | - | - | - | - | - | 1 |
| 15023 | F | 58 | Sarcoma | - | - | - | Y | - | - | - | - | - | - | 1 |
| 17853 | F | 43 | Sarcoma | - | - | - | - | - | - | - | Y | - | - | 1 |
| 16425 | F | 43 | Skin | Y | Y | - | - | Y | Y | - | Y | - | Y | 6 |
| 14405 | M | 27 | Testes | - | - | - | - | Y | - | - | - | - | - | 1 |
| 16613 | M | 29 | Testes | - | - | - | - | Y | - | - | - | - | - | 1 |
| 16435 | M | 67 | Sarcoma | Y | - | - | - | - | - | - | - | - | Y | 1 |
| 14984 | F | 53 | Uterus | - | Y | Y | Y | Y | - | - | - | - | Y | 4 |

Y indicates presence of metastatic site at each organ.
