# Supplementary material for: Encyclopedic tumor analysis for guiding treatment of advanced, broadly refractory cancers: results from the RESILIENT trial
Source: Oncotarget. 2019 Sep 24;10(54):5605–21. doi: 10.18632/oncotarget.27188 (PMC6771458; doi:10.18632/oncotarget.27188)
Supplement: Supplementary file 3 [file oncotarget-10-5605-s003.docx]

**Supplementary Table 4: Patient-wise details of prior lines of treatment and indications for ETA-guided treatments in the Intent to Treat (ITT) population**

| **ID** | **Age** | **Gender** | **Cancer Type / Organ** | **Prior Therapy Details** | | | | **Systemic Agents Received Previously** | | | **Indications and Agents in ETA-Guided Therapy** |
| --- | --- | --- | --- | --- | --- | --- | --- | --- | --- | --- | --- |
|  |  |  |  | **Total Lines** | **Surgeries** | **Irradiation** | **Systemic Lines** | **Cytotoxic** | **Targeted** | **Endocrine** |  |
| 9668 | 48 | F | Ovarian | 3 | 1 | 0 | 2 | Yes | No | No | Chemosensitivity: Cyclophosphamide  Chemosensitivity: Etoposide  Chemosensitivity: Methotrexate |
| 13770 | 50 | F | Ovarian | 11 | 2 | 0 | 9 | Yes | No | No | Chemosensitivity: Methotrexate  Chemosensitivity: 5-fluorouracil |
| 14133 | 41 | F | Breast | 2 | 0 | 0 | 2 | Yes | No | No | ESR1 (DGE): Exemestane AR (IHC): Bicalutamide Chemosensitivity: Vinorelbine |
| 14134 | 52 | F | Ovarian | 5 | 1 | 0 | 5 | Yes | No | No | Chemosensitivity: Etoposide  Chemosensitivity: Pemetrexed |
| 14135 | 57 | M | Hepatobiliary | 3 | 0 | 0 | 3 | No | Yes | No | Chemosensitivity: Gemcitabine  Chemosensitivity: Docetaxel TUBB3 (DGE): Docetaxel |
| 14137 | 35 | F | Neuroendocrine | 4 | 1 | 0 | 3 | Yes | No | No | Chemosensitivity: Oxaliplatin  EGFR, ERBB2 (DGE): Erlotinib  c-Kit, RET (DGE): Axitinib |
| 14152 | 24 | F | Colorectal | 3 | 0 | 1 | 3 | Yes | Yes | No | Chemosensitivity: Paclitaxel  Chemosensitivity: Vinblastine  Chemosensitivity: Cyclophosphamide |
| 14157 | 55 | M | Lung - NSCLC | 3 | 0 | 1 | 2 | Yes | Yes | No | Chemosensitivity: Cisplatin  Chemosensitivity: 5-fluorouracil  Chemosensitivity: Epirubicin  EGFR (DGE): Cetuximab |
| 14158 | 35 | F | Ovarian | 4 | 1 | 0 | 3 | Yes | No | No | Chemosensitivity: Cisplatin  Chemosensitivity: Etoposide |
| 14171 | 64 | M | Colorectal | 3 | 0 | 1 | 3 | Yes | Yes | No | Chemosensitivity: Paclitaxel  Chemosensitivity: Cisplatin  PDGFRA (DGE): Axitinib |
| 14172 | 55 | F | Colorectal | 7 | 2 | 0 | 5 | Yes | Yes | No | Chemosensitivity: Vinblastine  Chemosensitivity: Cisplatin |
| 14173 | 62 | M | Lung - NSCLC | 2 | 0 | 0 | 2 | Yes | No | No | Chemosensitivity: Vinblastine  FGFR1 (DGE): Pazopanib |
| 14183 | 36 | F | Breast | 3 | 1 | 0 | 3 | Yes | No | No | PTEN (SNV): Everolimus ESR (DGE): Exemestane |
| 14206 | 58 | F | Colorectal | 3 | 0 | 0 | 3 | Yes | Yes | No | Chemosensitivity: Pemetrexed  CYP17A1 (DGE): Abiraterone |
| 14207 | 35 | F | Breast | 5 | 1 | 1 | 3 | Yes | Yes | No | ERBB2 (CNA + DGE + IHC): Lapatinib, Chemosensitivity: Oxaliplatin  Chemosensitivity: Eribulin |
| 14226 | 32 | F | Colorectal | 2 | 1 | 0 | 2 | Yes | No | No | Chemosensitivity: Methotrexate  KRAS (WT): Cetuximab |
| 14232 | 56 | M | Melanoma | 4 | 1 | 1 | 3 | Yes | Yes | No | Chemosensitivity: Paclitaxel  Chemosensitivity: Topotecan  KIT (CNV+): Dasatinib KIT (SNV): Dasatinib |
| 14252 | 51 | M | Head and Neck | 5 | 2 | 2 | 4 | Yes | Yes | No | Chemosensitivity: Cyclophosphamide  Chemosensitivity: Methotrexate  KIT (CNV+, DGE): Axitinib FLT4, PDGFR, KDR (DGE): Axitinib |
| 14264 | 59 | F | Kidney | 4 | 1 | 2 | 1 | No | Yes | No | Chemosensitivity: Capecitabine  KIT (DGE): Axitinib |
| 14275 | 55 | F | Breast | 4 | 1 | 1 | 4 | Yes | No | Yes | Chemosensitivity: Vinorelbine, Chemosensitivity: Capecitabine |
| 14278 | 52 | M | Neuroendocrine | 3 | 1 | 0 | 2 | Yes | No | No | Chemosensitivity: Epirubicin  VEGF (DGE): Axitinib |
| 14295 | 55 | F | Head and Neck | 3 | 1 | 1 | 2 | Yes | No | No | Chemosensitivity: Cyclophosphamide  Chemosensitivity: Methotrexate  FLT4, PDGFRA, PDGFRB (DGE): Axitinib |
| 14307 | 49 | F | Breast | 17 | 1 | 2 | 14 | Yes | Yes | Yes | PIK3CA (SNV): Everolimus AR (IHC): Enzalutamide ER (IHC): Anastrozole Chemosensitivity: Eribulin |
| 14313 | 65 | F | Cervical | 2 | 0 | 1 | 1 | Yes | No | No | Chemosensitivity: Vinorelbine  Chemosensitivity: Cyclophosphamide  AR (IHC): Bicalutamide |
| 14355 | 39 | F | Breast | 5 | 1 | 1 | 4 | Yes | Yes | No | PIK3CA (SNV) Temsirolimus, VEGFA (DGE): Bevacizumab |
| 14363 | 53 | F | Breast | 2 | 0 | 0 | 2 | Yes | No | No | CCND1 (CNV+): Palbociclib  Chemosensitivity: Docetaxel |
| 14364 | 52 | M | Gastric | 1 | 0 | 0 | 1 | Yes | No | No | Chemosensitivity: Docetaxel  Chemosensitivity: Dacarbazine  PDGFR (DGE): Axitinib |
| 14402 | 69 | M | Gastric | 1 | 1 | 0 | 2 | Yes | No | No | Chemosensitivity: Paclitaxel  Chemosensitivity: Methotrexate  PDGFR (DGE) Axitinib |
| 14405 | 27 | M | Testicular | 3 | 0 | 0 | 3 | Yes | No | No | Chemosensitivity: Cyclophosphamide,  Chemosensitivity: Methotrexate,  Chemosensitivity: 5-fluorouracil |
| 14459 | 27 | F | Gastric | 2 | 1 | 0 | 1 | Yes | No | No | Chemosensitivity: Docetaxel  Chemosensitivity: Liposomal Doxorubicin |
| 14461 | 61 | F | Ovarian | 8 | 1 | 0 | 7 | Yes | No | No | Chemosensitivity: Mitoxantrone  VEGF (DGE): Bevacizumab  ER (IHC): Tamoxifen  AR (IHC): Bicalutamide |
| 14506 | 59 | F | Cervical | 2 | 0 | 0 | 2 | Yes | No | No | Chemosensitivity: 5-fluorouracil  PTEN (CNA-): Everolimus PIK3CA (SNV): Everolimus |
| 14507 | 43 | M | Lung - NSCLC | 2 | 0 | 0 | 2 | Yes | No | No | Chemosensitivity: Cisplatin Chemosensitivity: Capecitabine |
| 14522 | 43 | M | Colorectal | 4 | 2 | 1 | 2 | Yes | No | No | Chemosensitivity: Paclitaxel  Chemosensitivity: Methotrexate  FLT1 (DGE): Regorafenib |
| 14529 | 35 | M | Head and Neck | 4 | 1 | 1 | 3 | Yes | Yes | No | EGFR (CNV+): Cetuximab  JAK2 (CNV+): Ruxolitinib Chemosensitivity: Etoposide |
| 14533 | 57 | F | Head and Neck | 2 | 0 | 0 | 2 | Yes | No | No | Chemosensitivity: Cytarabine  Chemosensitivity: Etoposide |
| 14552 | 50 | M | Head and Neck | 2 | 0 | 0 | 2 | Yes | Yes | No | Chemosensitivity: Cisplatin  EGFR (DGE): Cetuximab  PDGFRA (DGE): Axitinib |
| 14553 | 42 | M | Bone | 2 | 0 | 1 | 2 | Yes | No | No | Chemosensitivity: Docetaxel Chemosensitivity: Gemcitabine |
| 14557 | 49 | F | Head and Neck | 4 | 0 | 1 | 4 | Yes | No | No | Chemosensitivity: Bortezomib  Chemosensitivity: Docetaxel  EGFR (DGE): Cetuximab |
| 14563 | 36 | M | Colorectal | 2 | 0 | 1 | 1 | Yes | No | No | Chemosensitivity: Paclitaxel  Chemosensitivity: Methotrexate KRAS (WT): Cetuximab |
| 14569 | 60 | M | Soft Tissue Sarcoma | 3 | 1 | 0 | 2 | No | Yes | No | Chemosensitivity: Cisplatin  Chemosensitivity: Vinblastine  PDGFR (DGE): Axitinib |
| 14573 | 46 | F | Head and Neck | 3 | 1 | 1 | 1 | Yes | No | No | EGFR (DGE) Cetuximab  Chemosensitivity: Irinotecan  Chemosensitivity: Oxaliplatin |
| 14581 | 60 | F | Breast | 6 | 1 | 1 | 5 | Yes | No | No | PIK3CA (SNV): Everolimus Her2 (CNV+): Lapatinib Dacarbazine: Chemosensitivity |
| 14644 | 55 | M | Head and Neck | 3 | 1 | 1 | 2 | Yes | No | No | Chemosensitivity: Paclitaxel  Chemosensitivity: Vinorelbine  EGFR (DGE): Afatinib ERBB2 (DGE): Afatinib |
| 14656 | 61 | F | Breast | 6 | 1 | 1 | 5 | Yes | No | No | VEGFA (DGE): Bevacizumab  PIK3CA (SNV): Everolimus  Her2 (CNV+): Lapatinib |
| 14668 | 49 | F | Breast | 4 | 1 | 2 | 3 | Yes | Yes | No | Chemosensitivity: Pemetrexed  Chemosensitivity: Everolimus  ERBB2 (DGE): Lapatinib |
| 14688 | 56 | M | Gastric | 4 | 0 | 0 | 4 | Yes | No | No | Chemosensitivity: Pemetrexed  Chemosensitivity: Carboplatin  PIK3CA (SNV): Everolimus |
| 14710 | 47 | M | Head and Neck | 4 | 2 | 0 | 2 | Yes | No | No | Chemosensitivity: Vinorelbine  Chemosensitivity: Topotecan |
| 14711 | 46 | F | Melanoma | 5 | 2 | 0 | 4 | Yes | No | No | Chemosensitivity: Vincristine  Chemosensitivity: Etoposide  PDGFRA, PDGFRB (DGE): Axitinib |
| 14726 | 54 | M | Head and Neck | 5 | 3 | 0 | 2 | Yes | No | No | Chemosensitivity: Cyclophosphamide  Chemosensitivity: Methotrexate |
| 14727 | 69 | F | Pancreatic | 3 | 0 | 1 | 2 | Yes | No | No | Chemosensitivity: Docetaxel  Chemosensitivity: Pemetrexed  Chemosensitivity: Temozolomide |
| 14752 | 61 | M | Colorectal | 10 | 0 | 6 | 5 | Yes | No | No | Chemosensitivity: Pemetrexed  EGFR (CNV+) Cetuximab  IGF1R (CNV+): Everolimus |
| 14844 | 47 | M | Head and Neck | 3 | 1 | 0 | 2 | Yes | No | No | CDKN2A (SNV): Palbociclib  EGFR (CNV+): Afatinib  Chemosensitivity: Methotrexate |
| 14851 | 39 | F | Colorectal | 3 | 1 | 0 | 2 | Yes | Yes | No | Chemosensitivity: Etoposide  Chemosensitivity: Methotrexate  KRAS (SNV): Regorafenib |
| 14858 | 67 | F | Hepatobiliary | 1 | 0 | 0 | 1 | Yes | No | No | ERBB2 (CNV+): Trastuzumab  Chemosensitivity: Vinblastine  Chemosensitivity: Etoposide |
| 14859 | 28 | M | Bone | 2 | 1 | 0 | 2 | Yes | No | No | Chemosensitivity: Eribulin Chemosensitivity: 5-fluorouracil |
| 14899 | 70 | M | Soft Tissue Sarcoma | 3 | 1 | 1 | 1 | No | Yes | No | Chemosensitivity: Oxaliplatin  Chemosensitivity: 5-fluorouracil |
| 14915 | 39 | F | Breast | 5 | 1 | 0 | 4 | Yes | No | No | Chemosensitivity: Vinorelbine  AKT2 (CNV+): Temsirolimus |
| 14984 | 53 | F | Soft Tissue Sarcoma | 2 | 1 | 0 | 1 | Yes | No | No | Chemosensitivity: Cisplatin  Chemosensitivity: Epirubicin |
| 15003 | 69 | F | Pancreatic | 2 | 1 | 2 | 1 | Yes | No | No | Chemosensitivity: Docetaxel  Chemosensitivity: Cyclophosphamide  FGFR4 (DGE) Regorafenib |
| 15023 | 58 | F | Soft Tissue Sarcoma | 2 | 1 | 0 | 1 | Yes | No | No | Chemosensitivity: Vinblastine  Chemosensitivity: 5-fluorouracil  PTEN (CNV-): Everolimus |
| 15071 | 46 | M | Lung - NSCLC | 5 | 0 | 1 | 4 | Yes | Yes | No | Chemosensitivity: Gemcitabine  PTEN (SNV): Everolimus  PIK3CA (CNV+): Everolimus EGFR (SNV): Osimertinib |
| 15132 | 45 | M | Head and Neck | 3 | 1 | 1 | 2 | Yes | No | No | Chemosensitivity: Cyclophosphamide  Chemosensitivity: Vincristine  Chemosensitivity: Etoposide EGFR (DGE): Afatinib |
| 15187 | 34 | F | Pancreatic | 1 | 0 | 0 | 1 | Yes | No | No | Chemosensitivity: Gemcitabine  Chemosensitivity: Vinorelbine  MET (CNV+): Crizotinib |
| 15205 | 46 | F | Breast | 6 | 2 | 1 | 4 | Yes | No | Yes | GPR124: Bevacizumab, ER (IHC): Fulvestrant, Chemosensitivity: Eribulin |
| 15223 | 42 | M | Head and Neck | 5 | 1 | 1 | 3 | Yes | No | No | Chemosensitivity: Gemcitabine  Chemosensitivity: Pemetrexed  PTEN (CNV-): Everolimus PIK3CA (SNV): Everolimus |
| 15292 | 58 | M | Hepatobiliary | 2 | 0 | 0 | 2 | Yes | No | No | Chemosensitivity: Docetaxel  PIK3CA (SNV): Everolimus |
| 15297 | 43 | M | Kidney | 1 | 0 | 0 | 1 | No | Yes | No | Chemosensitivity: Irinotecan  Chemosensitivity: 5-fluorouracil  VEGF (DGE): Bevacizumab |
| 15312 | 46 | F | Lung - NSCLC | 3 | 0 | 1 | 3 | Yes | Yes | Yes | Chemosensitivity: Etoposide  Chemosensitivity: Capecitabine  PIK3CA (SNV): Everolimus |
| 15329 | 51 | M | Head and Neck | 5 | 1 | 2 | 3 | Yes | Yes | No | Chemosensitivity: Paclitaxel  EGFR (DGE): Cetuximab |
| 15372 | 45 | F | Breast | 5 | 1 | 1 | 5 | Yes | Yes | Yes | Her2 (IHC): Lapatinib Her2 (IHC): Trastuzumab Chemosensitivity: Eribulin |
| 15426 | 62 | M | Kidney | 1 | 0 | 0 | 1 | No | Yes | No | Chemosensitivity: Docetaxel  AR (IHC): Bicalutamide |
| 15474 | 54 | F | Breast | 5 | 1 | 0 | 4 | Yes | No | No | Chemosensitivity: Gemcitabine  BRCA1 (SNV): Olaparib |
| 15540 | 72 | F | Breast | 9 | 2 | 1 | 6 | Yes | Yes | No | Her2 (): Lapatinib Chemosensitivity: Vinorelbine Chemosensitivity: Etoposide |
| 15549 | 32 | F | Hepatobiliary | 1 | 0 | 0 | 1 | Yes | No | No | Chemosensitivity: Paclitaxel  Chemosensitivity: 5-fluorouracil |
| 15570 | 49 | M | Head and Neck | 3 | 1 | 1 | 2 | Yes | Yes | No | Chemosensitivity: Gemcitabine  Chemosensitivity: Vinorelbine |
| 15610 | 39 | F | Breast | 9 | 1 | 1 | 7 | Yes | No | No | Chemosensitivity: Gemcitabine  Chemosensitivity: Dacarbazine  PDGFRA (SNV): Axitinib |
| 15623 | 44 | F | Cervical | 5 | 1 | 2 | 3 | Yes | No | No | CYP17A1 (DGE): Abiraterone  Chemosensitivity: Capecitabine  Chemosensitivity: Etoposide |
| 15637 | 62 | F | Ovarian | 11 | 2 | 1 | 8 | Yes | No | No | Chemosensitivity: Vinorelbine  ER (IHC): Fulvestrant |
| 15648 | 51 | M | Soft Tissue Sarcoma | 6 | 2 | 2 | 2 | Yes | Yes | No | Chemosensitivity: Docetaxel  Chemosensitivity: Gemcitabine  PDGFRB (DGE): Imatinib |
| 15723 | 53 | F | Breast | 4 | 0 | 0 | 4 | Yes | Yes | No | Her2 (IHC): Lapatinib  PIK3CA (SNV) Everolimus  Chemosensitivity: Etoposide |
| 15730 | 66 | F | Esophageal | 1 | 0 | 0 | 1 | Yes | No | No | TOP2A (DGE): Etoposide  KDR (DGE): Axitinib FLT, VEGF (DGE): Axitinib |
| 15777 | 59 | M | Pancreatic | 4 | 0 | 1 | 3 | Yes | Yes | No | VEGFA (DGE): Bevacizumab  TOP2A (DGE): Etoposide  ERBB2 (DGE): Afatinib |
| 15852 | 46 | M | Head and Neck | 7 | 3 | 2 | 2 | Yes | No | No | TUBB1 (DGE): Eribulin  Chemosensitivity: Cyclophosphamide  FLT4 (DGE): Axitinib |
| 15867 | 55 | M | Pancreatic | 4 | 2 | 1 | 1 | Yes | No | No | Chemosensitivity: Cyclophosphamide  Chemosensitivity: Methotrexate  ERBB2 (DGE): Trastuzumab |
| 15902 | 47 | F | Breast | 7 | 1 | 2 | 4 | Yes | No | No | PIK3CA (SNV): Everolimus  PDGFRB (SNv): Pazopanib |
| 15928 | 41 | M | Head and Neck | 3 | 1 | 1 | 1 | Yes | No | No | Chemosensitivity: Irinotecan  Chemosensitivity: Docetaxel  mTOR (SNV): Everolimus |
| 16093 | 42 | M | Head and Neck | 4 | 1 | 0 | 3 | Yes | Yes | No | Chemosensitivity: Dacarbazine  Chemosensitivity: Vinblastine  EGFR (CNV+): Cetuximab  AR (IHC): Bicalutamide |
| 16129 | 70 | M | Hepatobiliary | 3 | 1 | 0 | 2 | Yes | Yes | No | Chemosensitivity: Doxorubicin  Chemosensitivity: 5-fluorouracil  AR (IHC): Enzalutamide |
| 16335 | 42 | M | Head and Neck | 7 | 2 | 1 | 5 | Yes | Yes | No | Chemosensitivity: Decitabine  CDKN2A (CNV-): Palbociclib |
| 16425 | 43 | F | Skin | 6 | 2 | 1 | 3 | Yes | No | No | VEGFA (DGE): Bevacizumab  Chemosensitivity: 5-fluorouracil  Chemosensitivity: Etoposide |
| 16435 | 67 | M | Soft Tissue Sarcoma | 5 | 1 | 2 | 2 | Yes | No | No | TUBB6 (DGE): Cabazitaxel  CDK4 (SNV): Palbociclib  AR (IHC): Bicalutamide |
| 16486 | 56 | M | Gastric | 1 | 0 | 0 | 1 | Yes | No | No | Chemosensitivity: Doxorubicin  Chemosensitivity: Methotrexate |
| 16553 | 36 | M | Head and Neck | 3 | 0 | 2 | 2 | Yes | No | No | EGFR (CNV+): Bevacizumab  EGFR (CNV+): Erlotinib |
| 16590 | 65 | F | Ovarian | 4 | 1 | 0 | 3 | Yes | No | No | Chemosensitivity: Epirubicin  ER (IHC): Tamoxifen  AR (IHC): Enzalutamide |
| 16613 | 29 | M | Testicular | 2 | 0 | 1 | 1 | Yes | No | No | Chemosensitivity: Gemcitabine  Chemosensitivity: 5-fluorouracil  Chemosensitivity: Everolimus |
| 16714 | 54 | F | Gastric | 3 | 1 | 1 | 1 | Yes | No | No | Chemosensitivity: Temsirolimus  Chemosensitivity: Vinblastine  Chemosensitivity: Methotrexate |
| 16735 | 54 | F | Breast | 8 | 1 | 3 | 5 | Yes | No | Yes | PIK3CA (): Everolimus ERBB2 (DGE): Trastuzumab Chemosensitivity: Eribulin |
| 16740 | 62 | M | Neuroendocrine | 1 | 0 | 0 | 1 | Yes | No | No | TOP2A (DGE): Etoposide  TUBB (DGE): Cabazitaxel  FLT4 (DGE): Axitinib KDR, PDGFR (DGE): Axitinib |
| 16788 | 60 | F | Breast | 4 | 1 | 0 | 3 | Yes | No | No | Chemosensitivity: Topotecan  Chemosensitivity: Eribulin  ER (IHC): Exemestane |
| 16825 | 63 | M | Pancreatic | 2 | 1 | 0 | 1 | Yes | No | No | Chemosensitivity: Pemetrexed  Chemosensitivity: Doxorubicin  PIK3CA (SNV): Everolimus |
| 16827 | 57 | M | Lung - NSCLC | 3 | 1 | 1 | 1 | Yes | No | No | Chemosensitivity: Vinorelbine  Chemosensitivity: Mitoxantrone |
| 16863 | 49 | M | Head and Neck | 6 | 3 | 1 | 2 | Yes | No | No | Chemosensitivity: Vinblastine  Chemosensitivity: Everolimus  KDR (DGE): Axitinib PDGFRA, PDGFRB (DGE): Axitinib |
| 16972 | 50 | M | Head and Neck | 3 | 1 | 1 | 1 | Yes | No | No | Chemosensitivity: Methotrexate  Chemosensitivity: Vinblastine  PDGFRB (DGE): Axitinib |
| 17007 | 62 | M | Esophageal | 3 | 0 | 2 | 1 | Yes | No | No | TUBB4B (DGE): Vinblastine  PIK3CA (SNV): Everolimus  MET (CNA+): Crizotinib |
| 17088 | 41 | F | Breast | 1 | 0 | 0 | 2 | Yes | No | No | Chemosensitivity: Etoposide, Chemosensitivity: Dacarbazine  VEGFA (DGE): Bevacizumab |
| 17093 | 68 | F | Breast | 7 | 1 | 1 | 5 | Yes | No | Yes | Chemosensitivity: Daunorubicin, PIK3CA (SNV): Everolimus  AR (IHC): Bicalutamide  ESR (DGE, IHC): Fulvestrant |
| 17334 | 61 | F | Head and Neck | 3 | 1 | 1 | 1 | Yes | No | No | Chemosensitivity: Pemetrexed  Chemosensitivity: Cyclophosphamide |
| 17344 | 54 | F | Cervical | 3 | 1 | 0 | 2 | Yes | No | No | Chemosensitivity: Methotrexate  Chemosensitivity: 5-fluorouracil  PTEN (SNV): Everolimus PIK3CA (SNV): Everolimus |
| 17345 | 43 | M | Head and Neck | 5 | 1 | 1 | 3 | Yes | No | No | Chemosensitivity: Topotecan  Chemosensitivity: Doxorubicin  FLT4 (DGE): Axitinib KDR, PDGFRA, PDGFRB (DGE): Axitinib |
| 17346 | 50 | F | Ovarian | 7 | 0 | 0 | 7 | Yes | No | No | Chemosensitivity: Cyclophosphamide  TSC2 (SNV): Everolimus  AR (IHC) Bicalutamide  ER (IHC): Exemestane |
| 17365 | 33 | M | Kidney | 2 | 1 | 0 | 1 | Yes | No | No | Chemosensitivity: Epirubicin  ERBB2 (CNV+): Trastuzumab  PTEN (SNV): Everolimus |
| 17402 | 57 | M | Prostate | 3 | 2 | 0 | 1 | Yes | No | No | Chemosensitivity: Dacarbazine  Chemosensitivity: 5-fluorouracil  Chemosensitivity: Everolimus  AR (IHC): Bicalutamide |
| 17417 | 38 | M | Head and Neck | 4 | 0 | 1 | 3 | Yes | No | No | Chemosensitivity: Eribulin  Chemosensitivity: Capecitabine |
| 17459 | 28 | F | Colorectal | 3 | 0 | 0 | 3 | Yes | Yes | No | Chemosensitivity: Etoposide  Chemosensitivity: Methotrexate |
| 17463 | 45 | F | Breast | 2 | 0 | 0 | 2 | Yes | No | No | TOP2A (DGE), Chemosensitivity: Etoposide TUBB4B (DGE), Chemosensitivity: Eribulin VEGFA (DGE): Bevacizumab |
| 17488 | 35 | M | Head and Neck | 5 | 2 | 1 | 2 | Yes | No | No | Chemosensitivity: Methotrexate  Chemosensitivity: Everolimus PDGFRA, PDGFRB (DGE): Axitinib |
| 17508 | 47 | M | Colorectal | 3 | 1 | 0 | 2 | Yes | No | No | Chemosensitivity: Docetaxel  Chemosensitivity: Pemetrexed |
| 17531 | 34 | F | Colorectal | 3 | 2 | 0 | 1 | Yes | No | No | Chemosensitivity: Gemcitabine  Chemosensitivity: Docetaxel |
| 17540 | 57 | M | Head and Neck | 4 | 1 | 1 | 3 | Yes | No | No | Chemosensitivity: Methotrexate  Chemosensitivity: Vinblastine |
| 17577 | 70 | M | Pancreatic | 2 | 0 | 1 | 1 | Yes | No | No | Chemosensitivity: Doxorubicin  Chemosensitivity: Pemetrexed  FLT3 (CNV+): Sorafenib |
| 17714 | 75 | F | Breast | 3 | 1 | 0 | 2 | Yes | No | No | Chemosensitivity: Eribulin  FGFR (): Pazopanib  ER (IHC): Exemestane |
| 17733 | 45 | M | Head and Neck | 5 | 0 | 1 | 4 | Yes | No | No | Chemosensitivity: Gemcitabine  Chemosensitivity: Eribulin  PIK3CA (SNV): Everolimus |
| 17782 | 33 | M | Colorectal | 2 | 1 | 0 | 1 | Yes | No | No | Chemosensitivity: Gemcitabine  Chemosensitivity: Irinotecan  PDGFRB (DGE): Sunitinib |
| 17798 | 45 | M | Colorectal | 3 | 1 | 0 | 2 | Yes | No | No | TUBB4B (DGE): Paclitaxel  PIK3CA (SNV): Everolimus |
| 17853 | 43 | F | Soft Tissue Sarcoma | 3 | 1 | 1 | 1 | Yes | No | No | Chemosensitivity: Irinotecan  Chemosensitivity: Mitomycin  KDR, FLT1, FLT4 (DGE): Axitinib |
| 17901 | 59 | M | Lung - NSCLC | 1 | 0 | 0 | 1 | Yes | No | No | Chemosensitivity: Docetaxel  Chemosensitivity: Cyclophosphamide  EML-ALK fusion: Crizotinib |
| 17923 | 66 | M | Head and Neck | 4 | 0 | 2 | 4 | Yes | Yes | No | TUBB4A (DGE): Vinorelbine  TOP2A (DGE): Etoposide  CCND1 (CNV+): Palbociclib |
| 17984 | 47 | M | Head and Neck | 1 | 0 | 0 | 1 | Yes | No | No | Chemosensitivity: Etoposide  TOP2A (DGE): Etoposide Chemosensitivity: Methotrexate  PDGFRA, PDGFRB (DGE): Axitinib |
| 18011 | 38 | M | Head and Neck | 6 | 2 | 2 | 2 | Yes | No | No | Chemosensitivity: Vinorelbine  Chemosensitivity: Gemcitabine |
| 18038 | 28 | F | Breast | 4 | 0 | 1 | 3 | Yes | No | Yes | VEGFA (DGE): Bevacizumab TUBB4B (DGE): Eribulin AR (IHC): Bicalutamide ER (IHC): Tamoxifen |
| 18093 | 45 | M | Head and Neck | 5 | 0 | 2 | 3 | Yes | No | No | Chemosensitivity: Eribulin  Chemosensitivity: 5-fluorouracil  PDGFR (DGE): Axitinib |
| 18096 | 55 | M | Gastric | 6 | 2 | 1 | 3 | Yes | No | No | Chemosensitivity: Gemcitabine  Chemosensitivity: Pemetrexed  FGFR3 (CNV+) Pazopanib |
| 18102 | 46 | M | Head and Neck | 3 | 1 | 1 | 2 | Yes | No | No | Chemosensitivity: Irinotecan  Chemosensitivity: Vinorelbine  PDGFRA (DGE): Axitinib |
| 18246 | 60 | F | Cervical | 4 | 1 | 0 | 3 | Yes | No | No | Chemosensitivity: Etoposide  Chemosensitivity: Daunorubicin  Chemosensitivity: Cytarabine |
| 18435 | 53 | F | Hepatobiliary | 2 | 0 | 0 | 2 | Yes | No | No | Chemosensitivity: Vincristine  Chemosensitivity: Cyclophosphamide  Chemosensitivity: Etoposide |
| 18571 | 54 | M | Head and Neck | 3 | 0 | 1 | 2 | Yes | No | No | Chemosensitivity: Gemcitabine  Chemosensitivity: Vinorelbine  EGFR (CNV+): Afatinib |
| 18587 | 49 | M | Pancreatic | 6 | 1 | 0 | 5 | Yes | No | No | Chemosensitivity: Methotrexate  Chemosensitivity: Etoposide  Chemosensitivity: Dactinomycin |
| 18617 | 71 | F | Hepatobiliary | 1 | 0 | 0 | 1 | No | Yes | No | Chemosensitivity: Gemcitabine  Chemosensitivity: Vinorelbine  AR (IHC) Bicalutamide  VEGF (DGE): Bevacizumab |
| 18662 | 64 | F | Ovarian | 5 | 2 | 1 | 2 | Yes | No | No | Chemosensitivity: Methotrexate  Chemosensitivity: Capecitabine |
| 18679 | 37 | M | Head and Neck | 7 | 3 | 1 | 3 | Yes | No | No | Chemosensitivity: Methotrexate  Chemosensitivity: Etoposide  PDGFRA, PDGFRB (DGE): Axitinib |
| 18834 | 60 | M | Colorectal | 4 | 1 | 1 | 2 | Yes | Yes | No | Chemosensitivity: Paclitaxel  Chemosensitivity: Etoposide KRAS (WT): Cetuximab |
| 18848 | 31 | F | Breast | 5 | 2 | 0 | 3 | Yes | Yes | No | Chemosensitivity: Gemcitabine,  Chemosensitivity: Vinorelbine |

DGE: Differential Gene Expression (Overexpression); SNV: Single Nucleotide Variation; CNV: Copy Number Variation.
